# Algae as nutritional and functional food sources: revisiting our understanding

**DOI:** 10.1007/s10811-016-0974-5

**Published:** 2016-11-21

**Authors:** Mark L. Wells, Philippe Potin, James S. Craigie, John A. Raven, Sabeeha S. Merchant, Katherine E. Helliwell, Alison G. Smith, Mary Ellen Camire, Susan H. Brawley

**Affiliations:** 1 School of Marine Sciences, University of Maine, Orono, ME 04469 USA; 2 Integrative Biology of Marine Models, Station Biologique Roscoff, CNRS-Université Pierre et Marie Curie, Place Georges Teissier, 29680 Roscoff, France; 3 National Research Council of Canada, 1411 Oxford Street, Halifax, NS B3H 3Z1 Canada; 4 Division of Plant Sciences, University of Dundee (James Hutton Inst), Invergowrie, Dundee, DD2 5DA Scotland UK; 5 Plant Functional Biology and Climate Change Cluster, University of Technology Sydney, Ultimo, NSW 2007 Australia; 6 Department of Chemistry & Biochemistry, University of California-Los Angeles, 607 Charles E. Young Dr., East, Los Angeles, CA 90095-1569 USA; 7 Department of Plant Sciences, University of Cambridge, Downing St., Cambridge, CB2 3EA UK; 8 Marine Biological Association of the UK, Citadel Hill, Plymouth, PL1 2PB UK; 9 School of Food and Agriculture, University of Maine, Orono, ME 04469 USA

**Keywords:** Algal foods, Antioxidants, Arsenosugars, Experimental design, Microalgal supplements, Nutritional minerals, Omega-3-fatty acids, Polysaccharides, Sea vegetables, Vitamins

## Abstract

Global demand for macroalgal and microalgal foods is growing, and algae are increasingly being consumed for functional benefits beyond the traditional considerations of nutrition and health. There is substantial evidence for the health benefits of algal-derived food products, but there remain considerable challenges in quantifying these benefits, as well as possible adverse effects. First, there is a limited understanding of nutritional composition across algal species, geographical regions, and seasons, all of which can substantially affect their dietary value. The second issue is quantifying which fractions of algal foods are bioavailable to humans, and which factors influence how food constituents are released, ranging from food preparation through genetic differentiation in the gut microbiome. Third is understanding how algal nutritional and functional constituents interact in human metabolism. Superimposed considerations are the effects of harvesting, storage, and food processing techniques that can dramatically influence the potential nutritive value of algal-derived foods. We highlight this rapidly advancing area of algal science with a particular focus on the key research required to assess better the health benefits of an alga or algal product. There are rich opportunities for phycologists in this emerging field, requiring exciting new experimental and collaborative approaches.

**Electronic supplementary material:**

The online version of this article (doi:10.1007/s10811-016-0974-5) contains supplementary material, which is available to authorized users.

## Introduction

**Fig 1:**
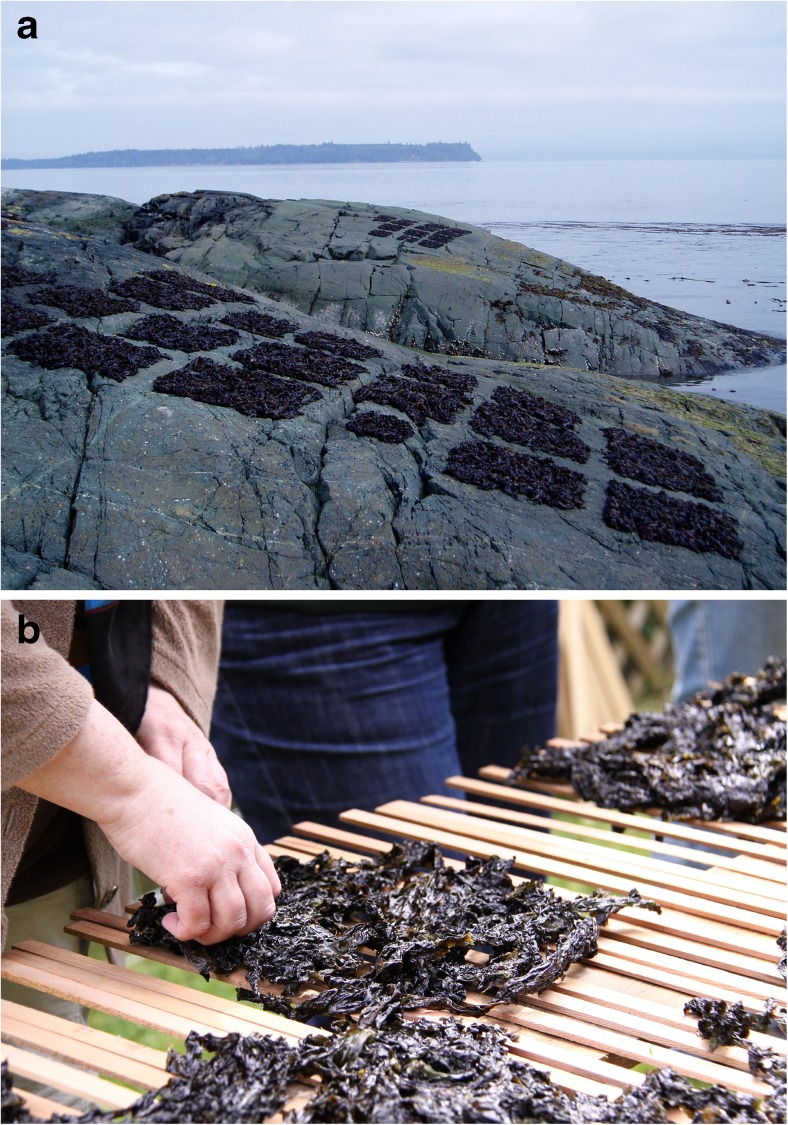
**a** *Pyropia* spp. being dried in squares in the intertidal zone by First Nations’ people at Pearce Island, British Columbia (2009). Harvesters would traditionally lay the seaweed out to dry on warm rocks while waiting for those fishing to return with the canoes (photo credit, Amy Deveau). **b** Checking the seaweed squares after transfer to cedar racks for final drying (photo credit, Victoria Wyllie-Echeverria)

Algae have been part of the human diet for thousands of years, based on archaeological evidence from 14,000 yBP in Chile (Dillehay et al. 2008) and early written accounts (e.g., in China, 300 A.D.; in Ireland, 600 A.D.; Newton 1951; Tseng 1981; Aaronson 1986; Turner 2003; Gantar and Svircev 2008; Craigie 2010). In North America, the Tsimshian First Nations’ people named the month of May for the time of year when they harvested the important food crop of *Pyropia* (Fig. 1). More contemporaneously, the global harvest of seaweeds in 2013 was estimated at US $6.7 billion, and over 95 % was produced in mariculture, with China and Indonesia being the top producers (FAO 2015). In addition to macroalgae, some microalgae are cultivated for foods and food additives (Switzer 1980; Jassby 1988; Fournier et al. 2005; Gantar and Svircev 2008; Chacón-Lee and González-Mariño 2010; FAO 2016). The FAO (2014) estimated that 38 % of the 23.8 million t of seaweeds in the 2012 global harvest was eaten by humans in forms recognizable to them as seaweeds (e.g., kelps, nori/laver), not counting additional consumption of hydrocolloids (e.g., agars, alginates, carrageenans) used as thickening agents in foods and beverages. Human consumption of algal foods varies by nation, with Japanese diets representing a recent (2010–2014) annual per capita consumption ranging from 9.6 (2014) to 11.0 (2010) g macroalgae day^−1^ (MHLW 2014). Overall, the trend towards increasing nutritional demand for algal products on a global basis stems from a greater focus on health and wider use of food additives.

In addition to their nutritional value, algae increasingly are being marketed as “functional foods” or “nutraceuticals”; these terms have no legal status in many nations but describe foods that contain bioactive compounds, or phytochemicals, that may benefit health beyond the role of basic nutrition (e.g., anti-inflammatories, disease prevention; Bagchi 2006; Hafting et al. 2012). The path from algal research to the launching of new food products or dietary supplements is strongly affected by industrial, regulatory, and nutritional considerations (e.g., see Borowitzka 2013a; Finley et al. 2014). The widespread interest in algal foods and/or their functional food potential is evident in numerous recent reviews (Warrand 2006; MacArtain et al. 2007; Kulshreshtha et al. 2008; Bocanegra et al. 2009; Mendes et al. 2009; Cottin et al. 2011; Harnedy and FitzGerald 2011; Holdt and Kraan 2011; Lordan et al. 2011; Pangestuti and Kim 2011; Stengel et al. 2011; Cornish et al. 2015; Hafting et al. 2015) and books (Rhatigan 2009; Mouritsen 2013; Tiwari and Troy 2015; Fleurence and Levine 2016). Many studies report the potential nutritional or bioactive content of different algae but many fewer studies quantify the bioavailability of nutrients and phytochemicals from algal foods. Our purpose is to review and assess what is known about different food components (i.e., proteins, polysaccharides, lipids, vitamins, minerals, and antioxidants, potential toxicants) in the context of improving knowledge about the efficacy of algal foods. There are rich opportunities for phycologists to collaborate with other scientists and clinicians in this emerging field from algal “prospecting” to defining nutritional value, bioaccessibility, and subsequent bioactivity, to the design and construction of mid-large cultivation systems for production of commercial-scale product.

## Digestion and bioavailability

In this article we use the term bioavailability, as defined by Carbonell-Capella et al. (2014) “as a combination of bioactivity and bioaccessibility,” where bioaccessibility refers to the release from the food matrix, transformations during digestion, and transport across the digestive epithelium, while bioactivity encompasses uptake into tissues, metabolism, and physiological effects. Because of the difficulties, both practical and ethical in terms of measuring bioactivity, the fraction of a given compound or its metabolite that reaches the systemic circulation (Holst and Williamson 2008) can be considered bioaccessible, but not necessarily bioactive. Most published evaluations of bioactivity of algal foods are based on short-term in vitro tests using algal extracts that frequently are of ill-defined composition and purity, so a clear understanding of their food value is highly constrained. Particularly lacking is information on the behavior of algal food components in the gut. For example, can the purported active metabolites identified in in vitro studies be transferred from the gut lumen into the body? Likewise, are observed in vivo biological effects the consequence of biological uptake or instead indirect outcomes stemming from improved functionality or composition of the intestinal microbiome? It is important then to consider the process of digestion and transformation in the human system.

Digestion refers to the physical and biochemical degradation of foods and the nutrients therein in preparation for absorption into the body. Digestion begins in the mouth with chewing, which reduces particle size and mixes food with saliva (Lovegrove et al. 2015). The predominant salivary enzyme is alpha (α)-amylase, which is specific for α(1→4) glucose linkages, and human salivary amylase is more active than that from other primates (Boehlke et al. 2015). Hardy et al. (2015) hypothesized that cooking to increase digestibility and sensory quality of starch-rich foods helped drive human evolution by providing more glucose to growing brains. Studies of the effect of human saliva on algae and specifically algal starch are lacking, however. The relative importance of salivary versus pancreatic amylase in starch digestion also is not clear (Lovegrove et al. 2015). Pepsin and the pepsinogens begin protein digestion in the stomach, aided by hydrochloric acid that denatures proteins and releases nutrients from the food matrix. Lipases produced in the mouth and stomach begin the process of digesting triacylglycerols. The stomach also releases intrinsic factor that is essential for vitamin B_12_ absorption in the small intestine. Gastric peristalsis further reduces food particle size, preparing macronutrients for additional chemical breakdown and absorption in the small intestine. The pancreas discharges a mixture of trypsin, chymotrypsin, carboxypeptidases, α-amylase, lipase, and other enzymes that respectively digest proteins and peptides, starches, triacylglycerols, and other compounds in the small intestine (Gropper and Smith 2013). The mixture of proteases, amylase, and lipase are collectively known as pancreatin; porcine pancreatin is often used to model human digestion in in vitro systems. The small intestine itself releases a variety of enzymes acting on peptides, amino acids, monoacyglycerols, disaccharides, and α(1→4) and α(1→6) linkages in oligosaccharides, dextrins, and polysaccharides such as starch. Micronutrients such as vitamins and minerals also are absorbed in the small intestine once they are solubilized from the food matrix. Fucoxanthin, a key algal carotenoid, may be better absorbed if other lipids are present (Peng et al. 2011).

Humans lack the ability to digest β(1→4) linkages in glucan polysaccharides, as in cellulose and hemicelluloses such as xyloglucan, and this indigestible material is referred to as dietary fiber. The undigested materials continue on to the large intestine (colon) where microbial co-metabolism ferments substrates such as non-starch polysaccharides, resistant starch, and oligosaccharides to short-chain fatty acids, and proteins into a wider variety of compounds. These bacterial-dependent enzymatic processes are not considered “digestion,” although the fermentation products can provide nutritional or functional benefits either by being absorbed and transported via the bloodstream or by shaping more healthful gut microbiomes and chemical conditions in the colon (MacFarlane and MacFarlane 2012). Indigestible, fermentable carbohydrates and sugar alcohols are referred to as FODMAP (fermentable, oligo-, di- mono-saccharides and polyols) (Gropper and Smith 2013). Algal proteins and carbohydrates that escape complete digestion in the small intestine may benefit humans by stimulating immune response indirectly via promotion of microbial responses (Cian et al. 2015). Dietary modulation of the colonic flora and the impact of bacterial fermentation products on human health are rapidly evolving areas of research (Duffy et al. 2015) and are likely to be especially important considerations in assessing the health benefits of algal-derived foods.

Not all human gut microbiomes have equal competencies, as algal polysaccharide fermentation differs among humans from different regions. The arsenal of polysaccharide-degrading enzymes exhibited in the common gut bacterium (*Bacteroides plebeius*) of Japanese people, but not Americans, appears to result from horizontal gene transfer (HGT) from *Zobellia galactanivorans* (Bacteroidetes), a marine bacterium inhabiting the surfaces of algae such as nori (Hehemann et al. 2010). HGT also may explain the presence of a gene cluster in Japanese gut *Bacteroides* that enables fermentation of alginates in brown algal cell walls (Thomas et al. 2012). Similarly, a small cohort of Spaniards possesses gut microbiomes with apparently HGT-provided porphyranases and agarases (Hehemann et al. 2012). Such striking differences emphasize the complex interactions among food customs, dietary history, and gut microbiomes that complicate study of the nutritional and functional benefits of algal foods (Paulsen and Barsett 2005; Costello et al. 2012; Gordon 2012; Nicholson et al. 2012).

The importance of assessing the biological availability of nutritional and functional food components cannot be underestimated. Bioavailability has critical relevance to both the proportional digestion and uptake of nutrients and functional food components, but also the degree of fermentation and nature of the host-microbial co-metabolism in the colon. While there exists a vast literature on the food content of microalgal and macroalgal foods and supplements, extrapolating these findings to assess their quantitative contribution to human health is more tenuous. The analytically determined concentration of constituents in food can differ, sometimes substantially, from that actually crossing from the digestive tract into the blood (i.e., the bioaccessible fraction). Moreover, current analytical approaches give even less insight to the complexity of interacting effects that regulate the bacterial flora of the colon, and hence the nature of fermentation products. Confounding issues stem from the food itself (e.g., the presence and nature of intact cell walls, soluble fiber characteristics, and the presence of other substances that may inhibit or facilitate the uptake of metabolites), the harvest season (e.g., altered metabolite and biomass composition, environmental variability of essential precursors, and anthropogenic factors), and the food preparation methods (Sensoy 2014). Analytical methods such as simulated gastrointestinal digestion (Moreda-Pineiro et al. 2011; Maehre et al. 2014), xenobiotic animal models, and molecular biological and genetic techniques can provide a sound basis for improved assessment of bioavailability; however, their use is not yet widespread in the study of foods of algal origin. As a consequence, and despite highly accurate and precise analytical determinations of food content, current knowledge of the nutritional or functional food value of algal products remains largely qualitative. The development of appropriate model systems and use of rigorous experimental design thus is essential in order to verify the bioavailability of nutritional and functional components of algae used in all foods.

## Proteins

**Fig 2:**
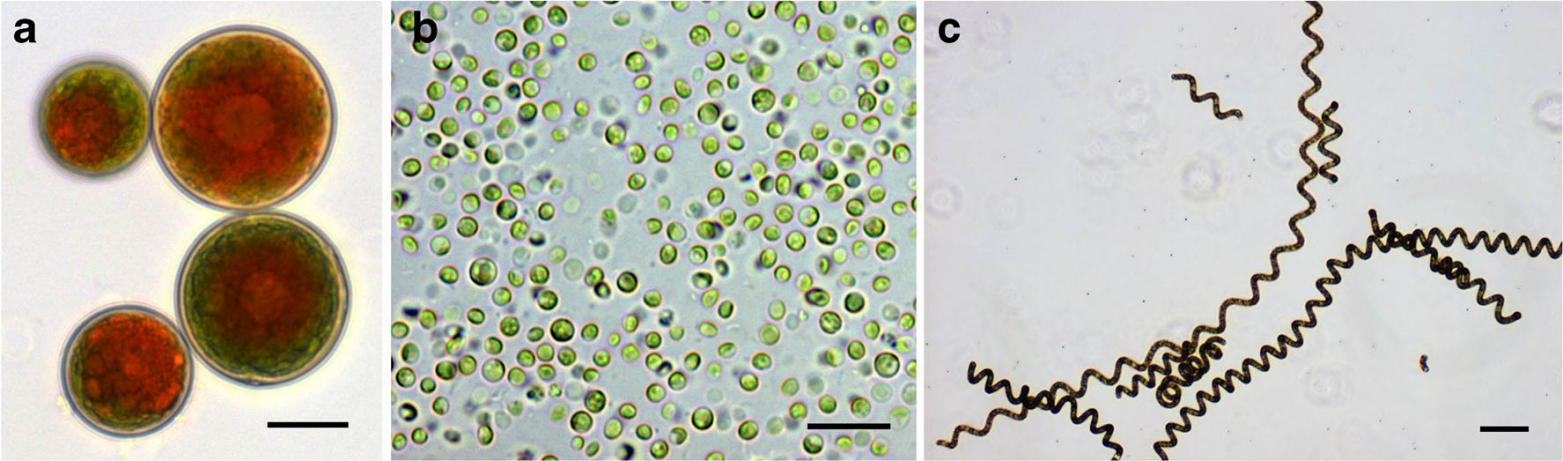
**a** *Haematococcus pluvialis* cells showing droplets of red astaxanthin within the cells; **b** *Chlorella vulgaris*; **c** *Arthrospira maxima* SAG 21-99 (also known as spirulina). *Scale bar* = 15 μm. (photo credits, Maria Zori)

Protein content differs widely across groups of algae (Online Resource 1). The filamentous cyanobacterium *Arthrospira platensis* (“spirulina”) and various commercial species of the unicellular green alga *Chlorella* (Fig. 2) contain up to 70 % dry wt protein; these microalgae also have an amino acid profile that compares well with egg, notably containing all of the essential amino acids (EAA) that humans cannot synthesize and must obtain from foods (Online Resource 2). Historically, “spirulina” was wild-harvested as a protein-rich whole food in many cultures outside Europe and North America (Gantar and Svircev 2008). Today, domesticated “spirulina” and *Chlorella* from several large producers have “GRAS” designations [Generally Recognized As Safe (FDA 2016)]. Large-scale production of both “spirulina” and *Chlorella* occurs throughout the world, and these well-domesticated crops are added to many foods to increase their protein and other nutritional contents (e.g., salad dressings, beverages, baked goods), and/or sold as protein supplements (e.g., Lubitz 1963; Ciferri 1983; Jassby 1988; Belay 1997; Gantar and Svircev 2008; Szabo et al. 2013; Safi et al. 2014).

Among the marine macroalgae, red and green algae [e.g., *Porphyra* spp. (“laver”), *Pyropia* spp. (“nori”), *Palmaria palmata* (“dulse”), *Ulva* spp. (“sea lettuce”)] often contain high levels of protein (as % dry wt) in contrast to lower levels in most brown algae (Online Resource 1; Dawczynski et al. 2007; Holdt and Kraan 2011; Pereira 2011; Taboada et al. 2013; Angell et al. 2016). During periods of nutrient limitation such as during the summer stratification of coastal waters, however, macroalgal protein content decreases, and the relative proportions of amino acids change (Online Resource 2; Galland-Irmouli et al. 1999; Johnson et al. 2014; Schiener et al. 2015). Historic harvesting times and current harvesting regimes usually occur at times when protein contents are favorable (e.g., Butler 1936; Black 1950; Turner 2003), but there is remarkably poor documentation of seasonal changes in protein content and amino acid profiles. Strong conclusions about nutritional content also depend upon good biological sampling (=simultaneously collected replicates) combined with appropriate laboratory analyses (=analytical replicates of each biological sample). Galland-Irmouli et al. (1999) analyzed one dulse blade/month (except August) from the Brittany coast with three technical replicates and found ∼15 % of dry mass as protein in a June blade while a November blade contained ∼23 % protein. A seasonal study (October 2010–October 2011, 3–8 months sampled/species) of protein content of four kelps (*Laminaria digitata*, *Laminaria hyperborea*, *Saccharina latissima*, *Alaria esculenta*) based on three technical replicates/species recently demonstrated an inverse relationship between protein content—higher in winter—and polysaccharide content—higher in summer, as well as clearly showing the higher protein content of *Alaria esculenta* (Online Resource 1) compared to the other kelps (Schiener et al. 2015). There is a pressing need for better replication of protein and amino acid analyses, as for all nutritional components in macroalgal studies, as well as better definition of the natural intertidal or commercial sites from which analyzed samples were obtained (N.B. Shuuluka et al. 2013 as example). Some new sea vegetable products will benefit from complementary food constituents (Woolf et al. 2011; see vProtein software).

Protein concentration in algae is often estimated using a total nitrogen-to-protein (NTP) conversion factor (6.25) based on the assumption that most N in the sample occurs as protein. This conversion factor, however, often over-estimates the protein content because of the presence of variable amounts of non-protein-N in the sample (Lourenço et al. 2002; Safi et al. 2013; Angell et al. 2016). For example, the conversion factor calculated for crude biomass for *Chlorella vulgaris* (walled) was 6.35, whereas it was 5.96 based on direct protein extracts (Safi et al. 2013). Similar studies of 19 tropical marine algae yielded even lower average factors of 4.59 (red algae), 5.13 (green algae), and 5.38 (brown algae) (Lourenço et al. 2002), perhaps related to seasonally lower N inputs to tropical surface waters. Zhou et al. (2012 [see her Tables 3–5]) reported similar findings. These conversion factors certainly will vary with season based upon varying amino acid composition, emphasizing the need for protein and amino acid studies to determine the seasonal optima for harvest among algal foods. Angell et al. (2016) argued for a new universal conversion factor, after finding a median nitrogen-to-protein value of 5 in a literature-based meta-analysis of 103 macroalgae; however, the range of values in their analysis was high (see their Fig. 4). The algae have polyphyletic origins and this, too, is reflected in the absence of a universal N to protein conversion factor.

In most analyses of amino acid composition in marine algae, glutamic acid, and aspartic acid represent the highest proportions of amino acids (e.g., Fleurence 1999b; Lourenço et al. 2002; Online Resources 1, 2; Holdt and Kraan 2011). These amino acids occur as protein constituents and as free amino acids or their salts. For humans, glutamate is the major component of the savory, the fifth basic taste called umami from its characterization in kelp (Ninomiya 2002; Mouritsen 2013). Glutamic acid content may decrease after several successive harvests of *Pyropia yezoensis* (nori; Niwa et al. 2008). Other amino acids (alanine and glycine) also contribute to distinctive flavors of some marine algae (e.g., see Holdt and Kraan 2011).

The non-proteinaceous amino acid taurine is especially abundant in marine red algae (e.g., ∼1–1.3 g taurine per 100 g DW of nori, Niwa et al. 2008). Although taurine is not an EAA for adults, it is a component of bile acids that complex and lower cholesterol in the bloodstream (Medeiros and Wildman 2015).

**Fig. 3:**
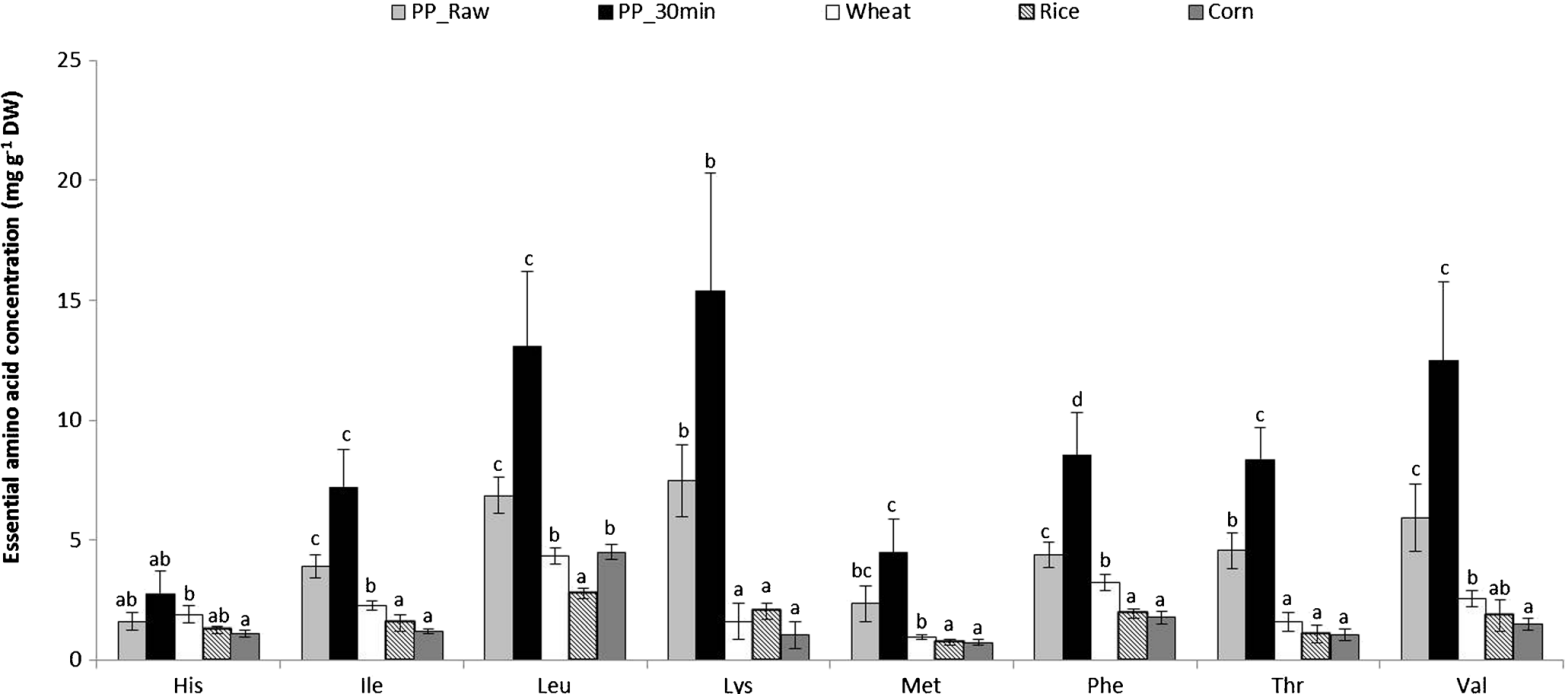
A comparison of essential fatty acids liberated from 1 g dry weight of *Palmaria palmata* (raw and boiled for 30 min) wheat, rice, and corn flours in simulated gastrointestinal digestion. The mean values ± 1 SD (*n* = 5) are shown in mg g^−1^. Significant differences between species (*p* > 0.05) are indicated by *different letters*. (Used with permission from Maehre et al. (2016))

In general, protein in most algae is digested less completely than reference proteins such as casein (a milk protein) in in vitro model systems containing digestive enzymes such as pepsin, pronase, and pancreatin, with evidence that this is due especially to inhibitory soluble fibers (e.g., Fujiwara-Arasaki et al. 1984; Fleurence 1999a; Urbano and Goni 2002; Marrion et al. 2003, 2005; Wong and Cheung 2003; De Marco et al. 2014). Inclusion of pre-analytical steps such as freezing, milling, digestion of crude sample with polysaccharide-digesting enzymes, and/or osmotic rupture of cells to free intracellular compounds is an active area of research (e.g., Harnedy and FitzGerald 2013; Safi et al. 2014; Ursu et al. 2014; and references therein). Importantly, a recent study (Maehre et al. 2016) with excellent biological and technical replication shows the beneficial effect of cooking on amino acid availability from dried dulse (Online Resource 3); however, cooking did not significantly increase the total amino acids measured from *Alaria* (Online Resource 3). Furthermore, Maehre et al. (2016) demonstrated that the apparent amino acid bioaccessibility from both raw and 30 min-boiled dulse was higher than from an equivalent dry weight of wheat, rice, or corn flour in a simulated in vitro gastrointestinal digestion model with analysis at each sequential digestive step (amylase/saliva buffer; pepsin/gastric buffer; pancreatin/duodenal buffer) (Fig. 3). Future research on microalgal and macroalgal protein bioavailability might incorporate measures such as the protein digestibility-corrected amino acid score (PDCAAS), which involves urinary and fecal determinations of N absorption in rats, as well as the FAO recommended replacement of PDCAAS by the digestible indispensable amino acid score (DIAAS) (Medeiros and Wildman 2015; Rutherfurd et al. 2015).

## Lipids

**Fig. 4:**
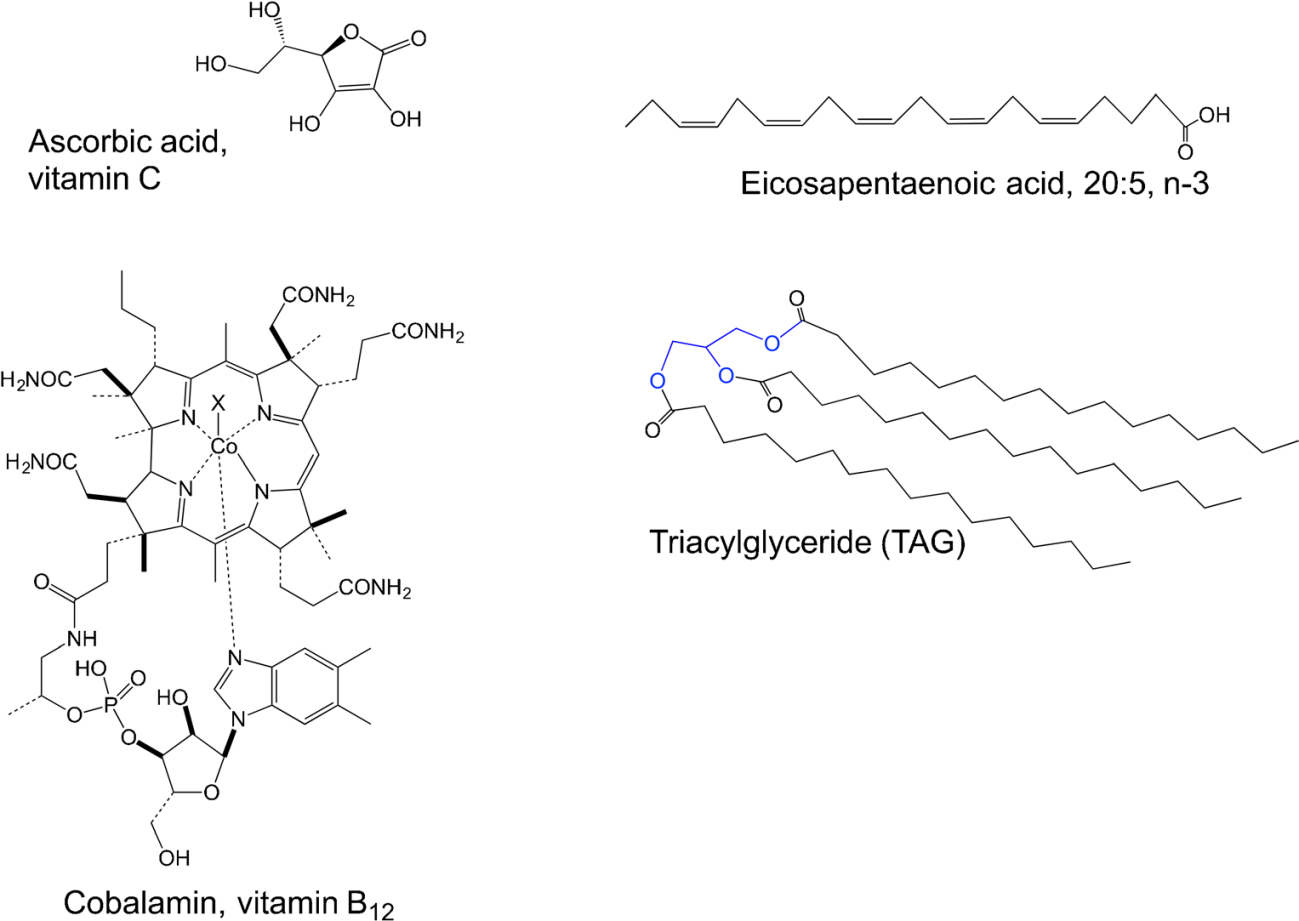
Structures of some key vitamins and lipids mentioned in review

Lipids are essential for all living organisms as components of membranes, energy storage compounds, and as cell signaling molecules (Eyster 2007). Although humans and other mammals synthesize lipids, some essential lipids must be obtained from dietary oils or fats. Phospho- and glycolipids, important for membrane function, contain a polar head group with two fatty acid chains, while the triacylglyceroles (TAGs), important energy stores in the cell, are non-polar (neutral) lipids containing three fatty acid chains (Fig. 4). Lipid membranes contain sterols such as fucosterol and β-sitosterol (Fahy et al. 2005) that also have reported health benefits (Arul et al. 2012). Embedded in algal lipid fractions are the nutritionally valuable carotenoid pigments that will be discussed in the “phytochemicals” section (below). TAGs have attracted great attention in recent years as a source for biodiesel, with some microalgae accumulating up to 40–60 % of their dry weight as TAGs (Georgianna and Mayfield 2012). However, marine macrophytes typically do not exceed 2–4.5 % dry wt as lipids, mainly as phospholipids and glycolipids (Holdt and Kraan 2011). Of these, the long-chain polyunsaturated fatty acids (PUFAs) and carotenoids are most noteworthy as functional foods (Holdt and Kraan 2011).

### Long-chain PUFAs

There are two general families of PUFAs: the linoleic acids (n-6 or omega 6 fatty acids) and the α-linolenic acids (n-3 or omega 3 fatty acids). Long-chain PUFAs comprise a substantial portion of marine algal lipids, with planktonic algae being the source of most omega fatty acids in fish. The most important of these PUFAs are the essential fatty acids (EFAs) eicosapentaenoic acid (EPA; 20:5 n-3) and docosahexaenoic acid (DHA; 22:6 n-3) along with their precursors α-linolenic acid (ALA; 18:3 n-3) and docosapentaenoic acid (22:5 n-3) (Cottin et al. 2011). The first product of ALA in the synthesis pathway to C20–22 PUFAs is stearidonic acid (SA, 18:4n-3), and this fatty acid can represent a significant portion of PUFAs in some edible macroalgae (sea vegetables) (Guil-Guerrero 2007). EPA is the predominant PUFA in many sea vegetables (Fig. 4), along with arachidonic acid (20:4 n-6), particularly in red algae (Norziah and Ching 2000; Wen et al. 2000; Ortiz et al. 2009) where EPA comprises up to 50 % of the total fatty acid content (e.g., *Palmaria palmata*, van Ginneken et al. 2011). Humans and other animals cannot convert ALA to EPA and DHA at required levels, so dietary sources of these EFAs are critically important for the health of humans (Cottin et al. 2011) and many animals (Li et al. 2009).

Numerous epidemiological and controlled interventional trials (N.B., the excellent reviews of Conquer and Holub 1996; Holub 2009; Cottin et al. 2011) support the health benefits to humans of DHA and EPA long-chain omega-3 fatty acids from fish oils and algal sources (mainly extracts). In contrast to most other algal food constituents, the bioaccessibility of DHA and EPA in algal-derived oils and extracts is well quantified for humans, ranging from ∼50 to 100 % depending on the matrix (Haug et al. 2011; Schuchardt et al. 2011). While clinical research to date strongly supports a nutritional need for oils that are enriched in DHA and EPA, there is more understanding about the bioactivity of DHA than of EPA (Conquer and Holub 1996; Holub 2009; Cottin et al. 2011). There is a considerable literature (Cottin et al. 2011) on the cardioprotective effects of DHA-containing TAG from *Crypthecodinium cohnii* (a dinoflagellate, Mendes et al. 2009) and *Schizochytrium* sp. (a thraustochytrid stramenopile, Li et al. 2009; Barclay et al. 2010), and as a consequence, infant formula, infant foods, and certain other food categories (dairy, bakery, eggs, and non-alcoholic beverages) and marketed nutritional products now are supplemented with algal-derived DHA. There is evidence that enhanced DHA intake may improve infant cognitive performance and enhance visual acuity (Jensen et al. 2005, 2010; Imhoff-Kunsch et al. 2011), although more recent data raises question about this linkage (Delgado-Noguera et al. 2015). There also is some understanding about the bioaccessibility of DHA in different algal products. Algal oil capsules based on a patented commercial source (Martek) and cooked salmon are reported to represent nutritionally equivalent sources of DHA (see in Cottin et al. 2011). A similar human trial showed that DHA from two different strains of *Schizochytrium* sp. (DHASCO-T and DHASCO-S) supplied in capsules generated equivalent dose-dependent DHA levels in plasma phospholipids and erythrocytes (Arterburn et al. 2008). Fortified snack bars also delivered equivalent amounts of DHA on a DHA dose basis (Arterburn et al. 2007). A systematic review of plant omega-3 fatty sources by Lane et al. (2014) concluded that further research on algal sources was warranted based on promising preliminary work.

Nonetheless, the relative health benefits of commercial algal supplements that tend to be DHA-rich versus natural fish oils that contain both DHA and EPA are uncertain. Cottin et al. (2011) found that “Recent evidence from randomized controlled trials has produced equivocal results. Heterogeneity of the studies in terms of dosage, duration, population target, sample size, as well as the relative amounts of EPA and DHA used in supplements could account for the variability of the results.” Even so, important trends stand out. While both EPA and DHA reduce TAG levels in humans (Wang et al. 2006; Bernstein et al. 2012), DHA appears responsible for the blood pressure and heart rate-lowering effect of fish oils (Valera et al. 2014). DHA also seems to be beneficial for endothelial and platelet function, although a direct role for EPA in regulating TAGs has not been ruled out. Algal DHA extracts can produce other cardiovascular protective effects in humans by altering plasma lipoproteins at reasonably small doses (2 g algal DHA day^−1^ over 4.5 months: Neff et al. 2011). The health benefits of algal DHA supplements for subgroups such as vegetarians, who otherwise may have low essential fatty acid intakes, remains a high research priority (Geppert et al. 2005; Cottin et al. 2011).

Fish oils also have demonstrated anti-inflammatory and insulin-sensitizing properties in vitro and in animal studies (Nauroth et al. 2010; Cottin et al. 2011); however, human trials often yield conflicting findings. Neither EPA nor DHA alone showed any effects on inflammation in double-blind trials with cystic fibrosis patients (Van Blervliet et al. 2008) or insulin sensitivity in human subjects, despite indications for potency in vitro (critically reviewed in Cottin et al. 2011). Without better quantification of the biological uptake of EPA or DHA, the reason for this discrepancy remains unknown.

Microalgae are the primary sources of DHA and EPA for zooplankton, fish, and other multicellular organisms, and these essential fatty acids (EFAs) become increasingly concentrated up the food web (e.g., Legezynska et al. 2014). Therefore, fish oils are rich in both DHA and EPA because they represent the trophic integration of DHA-rich flagellates and EPA-rich diatoms in the food web (Viso and Marty 1993). There is emerging evidence that ocean acidification, the result of changing coastal processes and increased atmospheric CO_2_, can negatively change the supply of these essential fatty acids to higher trophic levels (Rossoll et al. 2012). This and other factors affecting EFA production in algal assemblages will be an important area of future research (Chrismadha and Borowitzka 1994; Pasquet et al. 2014).

Concern over the sustainable supply of fish oils and the commercial dominance of algal-based DHA-only supplements has led to a large industry effort towards developing alternatives to fish oil-derived EPA (Zeller 2005). One example is Lovaza^TM^, a prescription pharmaceutical containing purified DHA and EPA synthesized from fish oils that reportedly have anti-hyperlipidemic properties (Weintraub 2014), although there are some negative indicators for this product (Spindler et al. 2014). A new promising biotechnological source of EPA has been proposed by Řezanka et al. (2010) from the Eustigmatophyceae *Trachydiscus minutus*; however, its commercial production is not developed yet. Other biotechnological production of EPA is provided by the diatoms *Phaeodactylum tricornutum* grown in tubular photobioreactors (Chrismadha and Borowitzka 1994) or *Odontella aurita* co-cultivated in raceway ponds with the red macroalga *Chondrus crispus* in France by the Innovalg company (Braud 2006). Commercial production of DHA and EPA is one of the main targets of producers and has benefited from the development of microalgal cultivation via fermentation technology (Branger et al. 2003; Barclay et al. 2013).

Several recent studies analyzed the constituent fatty acids of large numbers of red, brown, and green macroalgae from polar (Graeve et al. 2002, 20 species), temperate (Schmid et al. 2014, 16 species; McCauley et al. 2015, 10 species), and tropical (Kumari et al. 2010, 27 species; Kumar et al. 2011, 22 species) habitats, and, despite some species variability, red (Rhodophyta) and brown (Phaeophyceae) macroalgae had a high proportion of total FAs in EPA and arachidonic acid across latitudes, whereas the green (Chlorophyta) algae had low EPA (as % of total FA) but some DHA, and, were enriched in C18 LC PUFA. Phytoplankton contain more PUFA, as expected, when grown at low temperature (e.g., DHA in *Crypthecodinium*, Jiang and Chen 2000), and higher temperatures good for maximal biomass production can be lowered for as little as 12 h to induce maximal EPA content in the diatom *Phaeodactylum* (Jiang and Gao 2004).

**Fig. 5:**
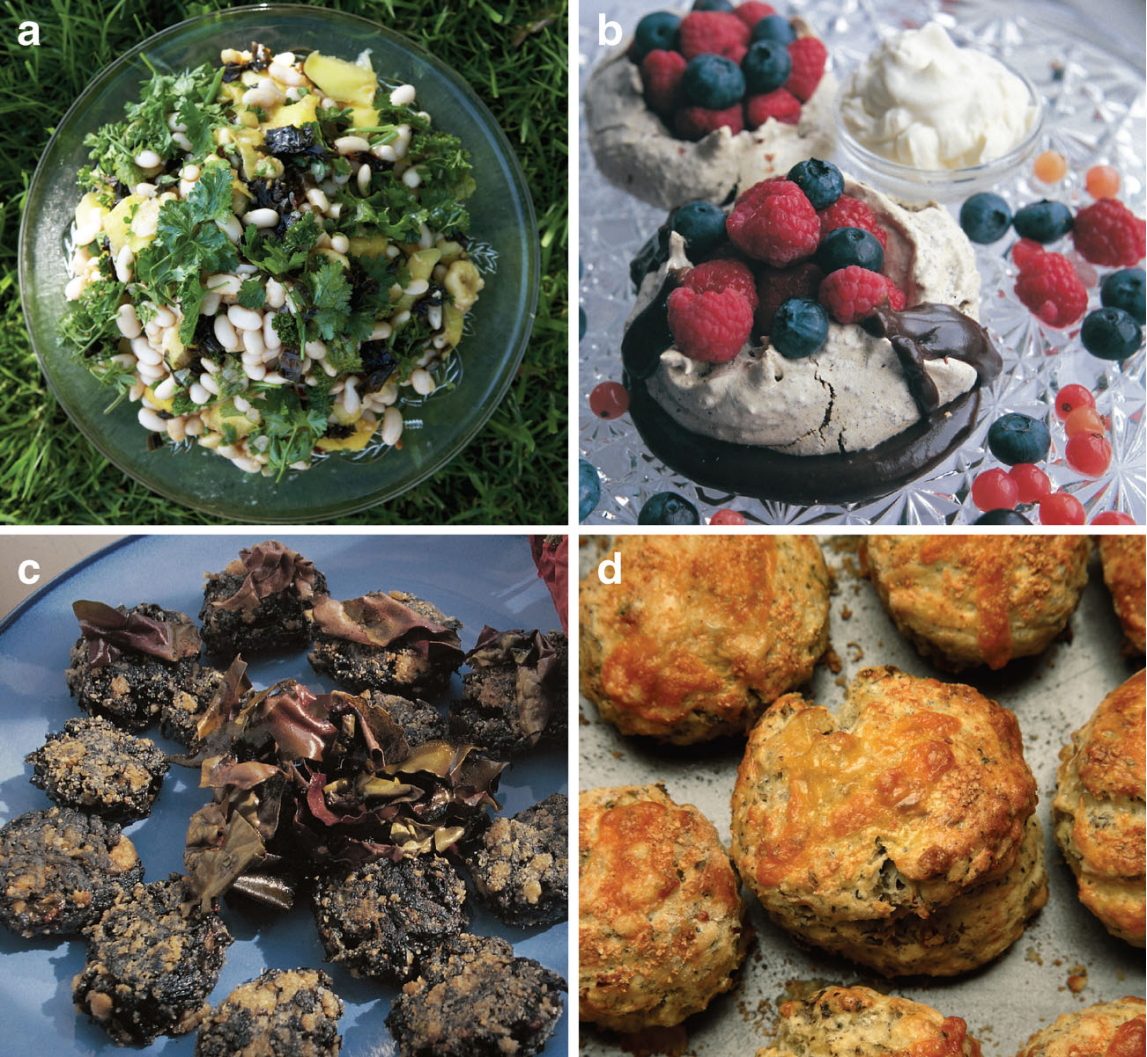
Sea vegetables used in European cuisine include marinated kelp (*Alaria esculenta*) in a cannelloni bean salad (**a**), laver/nori (*Porphyra umbilicalis*/*Pyropia yezoensis*) in chocolate molasses meringues (**b**), the traditional Welsh laver-bread cakes, with dulse (*Palmaria palmata*) crisps (**c**), and dulse-cheese scones (**d**). These additions add texture, protein, vitamins and minerals, and flavor. (Used with permission of Prannie Rhatigan from *The Irish Seaweed Kitchen*)

Whether omega-3 FA content can be manipulated by the timing of wild harvest or grow-out of sea vegetable crops in winter to increase EFA of whole foods needs much more work. Marine macrophytes generally contain low total lipid contents, so their comparative value as a food energy source likely is small (Holdt and Kraan 2011; Maehre et al. 2014), and at realistic daily consumption levels (e.g., 8 g dry wt., Blouin et al. 2006), even red algae such as *Porphyra umbilicalis* (laver) and *Palmaria palmata* (dulse) (Fig. 5) that have a high proportion of their total fatty acids as EPA (Mishra et al. 1993; Graeve et al. 2002; Blouin et al. 2006; Schmid et al. 2014) will not meet dietary recommendations for daily EFA alone (Blouin et al. 2006), although higher levels of macroalgae can support EFA needs for animal aquaculture feeds (Mulvaney et al. 2015; Wilke et al. 2015). Thus, relevant growth conditions should be manipulated for promising macroalgae in the laboratory to see if further increases are possible; unialgal cultures will be important because recent PUFA studies of green algae in a “green tide” in the Gulf of Finland showed that the high EPA content was largely due to epiphytic diatoms (Gubelit et al. 2015).

The bulk of research on omega-3 long chain PUFAs in microalgae and sea vegetables has been empirical, testing differences among species under different growth conditions. It will be important to understand their biosynthetic pathways and metabolic controls, and the increasing availability of microalgal genomes should provide excellent opportunities in this goal. One recent example is five genes functionally characterized in the haptophyte *Emiliania huxleyi* that are predicted to underpin omega-3 LC-PUFAs synthesis (Sayanova et al. 2011). Additional putative genes for functionally redundant pathways for the synthesis of omega-3 EPA and DHA were also annotated in the *E. huxleyi* pan genome sequence (Read et al. 2013).

Further investigation to produce biomass or extracts of sea vegetables containing EPA at a range of doses compatible with functional foods would enable research to examine the protective effects of consuming this source of long chain n-3 PUFA. Such trials could provide clear evidence for the clinical therapeutic potential of consuming EPA rich macroalgae in combination with supplementation of microalgal DHA.

### Sterols

Algae vary in their total sterol content and in the variety of sterols present (Holdt and Kraan 2011). Older analytical techniques may have misidentified algal sterols as cholesterol since their structures are similar (Pereira et al. 2016). Fucosterol occurs in many algae, especially red and brown macroalgae (Pereira et al. 2016), and this compound may have value in treating complications of diabetes and hypertension, as well as other major health concerns (Abdul et al. 2016). However, like other studies of algal foods, the linkages are implied but little is known about the actual in vivo effects of fucosterol when algae are consumed by humans. Nonetheless, as for long-chain PUFAs, understanding the seasonal, environmental, and species-specific factors that alter the abundance and composition of algal sterols, such as in the recent studies in Antarctic seaweeds (Pereira et al. 2016), will be fundamental to understanding their potential effects in human diets.

## Polysaccharides

Polysaccharides are used for energy storage and as structural elements in marine algae and terrestrial plants. Humans possess enzymes that degrade algal starches to mono-and di-saccharides for transport across the gut lumen, but generally cannot digest the more complex polysaccharides, as was first recognized more than a century ago (Saiki 1906). These resistant polysaccharides, known as dietary fiber, may be fermented in the large intestine to varying degrees depending on the enzymatic competence of the microbiome (Terada et al. 1995; Michel and MacFarlane 1996; Hehemann et al. 2010; Cian et al. 2015). Algal cell walls differ from those of terrestrial plants as they contain uncommon polyuronides and polysaccharides that may be methylated, acetylated, pyruvylated, or sulfated (Paulsen and Barsett 2005; Pal et al. 2014; Rioux and Turgeon 2015; Stiger-Pouvreau et al. 2016). It is fair to say that algal polysaccharides are the most widely, and often unknowingly, consumed food of algal origin. Small amounts are incorporated into beverages, meat and dairy products, and fillers (Cofrades et al. 2008; Gupta and Abu-Ghannam 2011a, b; Griffin 2015) at levels generally deemed to be beneficial and safe by regulatory agencies (extensively reviewed in Mabeau and Fleurence 1993; MacArtain et al. 2007; Watson 2008; Holdt and Kraan 2011; Barlow et al. 2015; Fleurence and Levine 2016).

Edible macroalgae contain unusually high amounts of dietary fiber, ranging from 23.5 % (*Codium reediae*) to 64.0 % of dry weight in *Gracilaria* spp., values that frequently exceed those for wheat bran (Ruperez and Saura-Calixto 2001; McDermid et al. 2005; Benjama and Masniyom 2012). The nomenclature of food-derived fiber is confusing because there is no consensus on its definition among scientists and regulatory agencies. *Dietary fiber*, considered a nutrient in the USA under the Nutrition and Education Act of 1990 (Thomas.loc.gov/ H.R. 3562.ENR), comprises “nondigestible carbohydrates and lignin that are intrinsic in intact plants.” Some fraction of this human-inert matter is considered by some as *Functional fiber*; that fraction of isolated, non-digested carbohydrates having apparent beneficial physiological effects beyond nutrition in humans (Institute-of-Medicine 2005; Medeiros and Wildman 2015). In this case, *Total fiber* is the sum of *dietary* and *functional* fiber (Institute-of-Medicine 2005; Medeiros and Wildman 2015). In contrast, the European Food Safety Authority, following the CODEX Alimentarius Commission definition of *dietary* fiber (Jones 2014), acknowledges that benefits beyond nutrition can occur but does not formally distinguish *functional* from *dietary* fiber because no analytical methods exist for this differentiation (EFSA 2010). Regardless of these semantics, non- or partially fermented fiber that generates physiological benefits, through either physical or chemical pathways, meets the definition of *dietary* fiber (Jones 2014).

“Soluble fiber” comprises 52–56 % of total fiber in commonly used green and red macroalgae and 67–85 % in brown macroalgae (Lahaye 1991). Much of it can be fermented to short-chain fatty acids (SCFAs) such as acetate, propionate, and butyrate (see Table 1 in Michel and MacFarlane 1996; Cantarel et al. 2012) which both nourish the epithelia of the large intestine and offer other benefits to the host (Terada et al. 1995; Michel and MacFarlane 1996). For example, acetate and propionate are transported in the blood to many organs where they are oxidized for energy or utilized in signaling to help regulate aspects of energy homeostasis and immune function (reviewed by Nicholson et al. 2012). The fermentation process and SCFA products also nourish and modify the microbial consortia in the large intestine, thereby exerting prebiotic effects and influencing digestive outcomes (e.g., Fernando et al. 2008; O’Sullivan et al. 2010; Cian et al. 2015). Investigating the coupling of algal (and other) polysaccharides to the health of intestinal microbiomes and their animal and human hosts is an active and needed area of research (Bäckhed et al. 2005; Hehemann et al. 2010; Cantarel et al. 2012). These beneficial responses may include reduced risk of diabetes, hypertension, and cardiac heart disease (Institute-of-Medicine 2005). However, the complexity of interactions among functional and dietary fiber and the intestinal microbiome challenges efforts to demonstrate the functional food and biomedical benefits of algal polysaccharides (de Jesus Raposo et al. 2015; Dhargalkar 2015).

The evidence for bioactivity of algal polysaccharides derives largely from in vitro experiments using isolated oligomers/polymers, with fewer data on testing any whole alga in animal or human trials. Compositional analysis of *Chlorella* and similar microalgae began more than 60 years ago, and an impressive number of biological processes are now reported to be influenced by ingestion of whole algae or polysaccharide extracts as food or supplements (Pulz and Gross 2004; Plaza et al. 2009; Chacón-Lee and González-Mariño 2010; Lordan et al. 2011; Vo et al. 2011). Microalgal genera (Fig. 2) commonly considered as beneficial dietary supplements include *Chlorella*, *Arthrospira (*spirulina), *Dunaliella*, *Haematococcus*, *Scenedesmus*, *Aphanizomenon*, *Odontella*, and *Porphyridium*, with species of *Chlorella* being recognized as particularly rich in polysaccharides (Chacón-Lee and González-Mariño 2010). This putative bioactivity includes anticancer properties, cytokine modulation, anti-inflammatory effects, macrophage activation, and inhibition of protein tyrosine phosphatase (Hasegawa et al. 1997; Cheng et al. 2004; Kralovec et al. 2005; Sheng et al. 2007; Hsu et al. 2010). Algal polysaccharide extracts can possess strong immunomodulating effects both in vitro and in vivo (Watanabe and Seto 1989; Pasco and Pugh 2010; Suárez et al. 2010). Kwak et al. (2012) observed an immunostimulatory effect in 30 Korean volunteers fed 5 g day^−1^ *Chlorella* vs. placebo in a double-blinded 8-week trial. Acidic polysaccharide extracts from *Chlorella pyrenoidosa* have been patented (Chlon A and Respondin^TM^) as potentially useful anti-tumor and immunostimulating supplements (Umezawa and Komiyama 1985; Komiyama et al. 1986; Kralovec 2005; Kralovec et al. 2005). Even so, the molecular structures responsible for such observed physiological functions are poorly understood because of fragmentary and sometimes conflicting information on the chemistry of these large, highly complex cell wall polymers (Řezanaka and Sigler 2007). Research also has focused on strikingly few algal species, leaving a broad window of opportunity for more expansive assessment of potential sources of bioactive compounds (Admassu et al. 2015).

The study of extracted polymer sub-fractions of structural polysaccharides provides a useful exploratory tactic for assessing the potential functional benefits of consuming macroalgal foods, and it establishes a quantitative means to determine the seasonal or environmental effects on food quality (Stengel et al. 2011; Mak et al. 2013). The predominant algal polysaccharides are the alginates in brown macroalgae, and the sulfate-esterified polysaccharides of macro- and microalgae that are widespread in red, brown, and green seaweeds (Aquino et al. 2005; Popper et al. 2011). The cellular quantities and compositions of these polysaccharides vary among species and with seasonal and environmental changes (Bourgougnon and Stiger-Pouvreau 2011; Mak et al. 2013).

### Alginate

Alginate is the major polysaccharide of brown algae, comprising 14–40 % of its dry mass (cf. Ramberg et al. 2010), and was first isolated in 1881 as algin from kelp (*Laminaria* sp.) by E. C. C. Stanford. The direct consumption of brown algae as human food is of long standing (Tseng 1981; Druehl 1988; Dharmananda 2002; McHugh 2003). The purported beneficial effects of alginate include its ability to absorb toxins, decrease cholesterol uptake, alter the colonic bacterial profiles, and generate SCFAs (Brownlee et al. 2005). The metal chelating abilities of alginates makes them valuable scavengers of toxic elements in the human gut, but this scavenging also may lead to nutritional deficiencies of essential di- or polyvalent trace metals (Hollriegl et al. 2004; Brownlee et al. 2005). Most studies have investigated the effects of polysaccharide extracts rather than consumption of intact seaweeds. Although the extent of alginate dissociation from algal cell walls after ingestion is not well studied, there is little or no digestion of sodium alginate from *Ascophyllum nodosum* in humans (Percival and McDowell 1967; Painter 1983; Aarstad et al. 2012). Dietary alginates also provide a sense of satiety and so have been explored as a weight control measure, although there remains uncertainty about its efficacy in this role (Yavorska 2012).

### Sulfated heteroglycans—ulvans

The abundant, heavily sulfated ulvans are extracted from members of the Ulvales. They are the best studied of the green seaweed polysaccharides, in part because the high production of *Ulva* spp. in eutrophic coastal waters has sparked research for new uses of these algae (Alves et al. 2013). Ulvans owe their bioactive properties to their unusual hydrophilic polyanionic features and structural analogies with animal glycosaminoglycan regulators (dermatan sulfate, heparin/heparin sulfates) and L-rhamnose specific lectins in humans. The reported bioactivities of ulvan extracts in vitro include antibacterial, anticoagulant, antioxidant, antiviral, anti-tumor, anti-hyperlipidemic, and immunoregulatory effects (Kaeffer et al. 1999; Yu et al. 2003; Mao et al. 2006, 2008; Leiro et al. 2007; Zhang et al. 2008, 2010; Lee et al. 2010; Holdt and Kraan 2011; Matloub et al. 2013).

Although the ingestion of green macroalgae by humans is rather widespread, the potential health benefits of food supplements of native ulvans or their chemically modified derivatives, let alone the direct consumption of the whole algae, are not well understood (Taboada et al. 2010; Wijesekara et al. 2011). Fermentation of *Ulva* and ulvan by human colonic bacteria was slight (16.6 and 8.9 % of organic matter, respectively) (Durand et al. 1997), indicating that they would be poor sources of SCFA production in the colon (Bobin-Dubigeon et al. 1997). However, these results cannot be generalized because only two individuals provided the bacterial inocula, and their prior dietary history relating to algal foods was unknown. A cautionary note here though is that *Ulva* can be rich in free sulfate which is readily converted to sulfide during fermentation, so consumption of more than 20 g day-*1* of the dry, unprocessed seaweed may have adverse (and odiferous) health effects (Durand et al. 1997).

### Sulfated galactans—carrageenans

Red algal polysaccharides include the nutritionally important floridean starch, and their sulfated galactans are known to initiate or modulate a large number of biological activities of significance to human health (Prajapati et al. 2014). The most studied are the sulfated agarocolloids and the carrageenans derived from macroalgae in the orders Gelidiales, Gigartinales, and Gracilariales. Anti-viral activities include those against herpes simplex, herpes zoster, dengue-2, vaccinia, rabies, and vesicular stomatitis virus with patents and some commercial projects resulting (Richards et al. 1978; Baba et al. 1988; Vedros 1993; Bourgougnon 2003; Eccles et al. 2010; Talarico et al. 2011; Levendosky et al. 2015; Luo et al. 2015). Whether consumption of the relevant red algae or their extracts in foods is protective against viruses does not appear to be known and deserves study. Carrageenan extracts that are depolymerized to various degrees have potential as tumor inhibitors and as immunostimulators in cancer therapy. Oligomers from acid hydrolyzed κ-carrageenan injected into mice increased macrophage phagocytosis and stimulated several immune-related markers while significantly inhibiting the growth of sarcoma S180 cells (Yuan et al. 2006). Phosphorylation or further sulfation of these oligomers increased the activity of natural killer cells, the cytotoxic lymphocytes critical to immune system function (Yuan et al. 2011). Similarly, transplanted human sarcoma S180 tumors were inhibited significantly in mice fed fractionated λ-carrageenan extracts of *Chondrus ocellatus* (200 mg kg^−1^ daily) (Zhou et al. 2004). Although seaweeds containing carrageenans act as prebiotics when supplied as supplements in both poultry and rat diets (Kulshreshtha et al. 2014; Liu et al. 2015), the potential for sulfated galactans from algae to benefit human health remains to be established.

Carrageenans have the potential to be harmful (Tobacman 2001). Carrageenan extracts generate proinflammatory agents in mice (Hansra et al. 2000), and the resulting public health concerns have led to several actions regarding carrageenans in food products (Watson 2008). Carrageenan is prohibited in the EU for use in infant formulas, and, although it is permitted in the USA, it must be of high molecular mass (i.e., >100 kDa with <5 % of 50 kDa fragments). High doses of low molecular mass carrageenan cause ulceration in the guinea pig colon (Watson 2008) and lead to marked increases in the chemokine interleukin-8 and B-cell CLL/lymphoma 10 activities in the normal human colonic mucosal epithelial NCM460 cell line (Bhattacharyya et al. 2010). Oral introduction of undegraded λ-κ carrageenan in drinking water of 12-week-old mice also caused significantly higher peak glucose levels in the blood, leading to concern that carrageenan-induced insulin resistance might contribute to human diabetes (Bhattacharyya et al. 2012). However, a comprehensive examination of in vivo dietary κ-carrageenan effects in rats revealed no effects on blood glucose (Weiner et al. 2007). More recent appraisals of carrageenans as food additives could find no hazards to human health as they are currently used (McKim 2014; Weiner 2014; Barlow et al. 2015; Weiner et al. 2015). The potential benefits and negative effects of including algae or their refined products in the diet require continuing research on a case-by-case basis.

### Beta-(1-3)-glucans—laminarans

The main polysaccharides after the alginates in brown algae include β-glucans (laminarans), cellulose, and heteroglycans, the first being an energy reserve while the others are structural components of the cell wall, fitting the definitions of dietary fiber (Jones 2014). The concentrations and composition of the β-glucans vary substantially with season and growth rates (Rioux et al. 2009). The most studied β-glucans are those from cereals and fungi, but these differ significantly in structure from those of algal origin (Rioux et al. 2010). The biological responses elicited by algal β-glucans depend strongly on details of their primary structures (Bohn and BeMiller 1995; Mueller et al. 2000; Williams et al. 2005; Hofer and Pospíšil 2011). For example, brown algal M-series laminaran showed significant hepatoprotective effects when ingested orally by rats (Neyrinck et al. 2007). The protection was organ specific and appeared to act via the Kupffer cells in the liver, thereby establishing an immunoregulatory function of this orally ingested functional fiber. These and other biological effects of β-glucans have been reviewed (Novak and Vetvica 2008; Ramberg et al. 2010; Lehtovaara and Gu 2011; Kadam et al. 2015), and certain cautions have been expressed about the functional effects of soluble and particulate forms of these compounds (Young and Castranova 2005; Hofer and Pospíšil 2011).

### Sulfated fucose-containing polysaccharides—fucoidans

The fucoidans are a subset of marine fucose-containing polysaccharides (FCPs) found in brown algae (Painter 1983) that are now attracting widespread interest (Shanmugam and Mody 2000; Berteau and Mulloy 2003; Kusaykin et al. 2008; Li et al. 2008; Pomin and Mourão 2008; Courtois 2009; Pomin 2009, 2012; Fitton 2011; Jiao et al. 2011; Kim and Li 2011; Kim and Wijesekara 2011; Wijesinghe et al. 2011; Wijesinghe and Jeon 2012). Double-blind clinical trials with fucoidan extracts show anti-aging effects on skin and other benefits in cosmetic applications (Fitton et al. 2015). A common source of FCPs used in experimental studies is *Fucus vesiculosus*, but fucoidans also are found in edible species such as *Cladosiphon okamuranus*, *Saccharina japonica* (*as Laminaria japonica*), and *Undaria pinnatifida* (Fitton 2011). The highly sulfated nature and molecular weights of FCPs appear to be responsible for many demonstrated biological activities in vitro (Croci et al. 2011; Ustyuzhanina et al. 2014). The FCP structures are species-dependent and can be modified by environmental variables and the developmental status of the seaweed fronds, all of which can affect their bioactivities (Honya et al. 1999; Zvyagintseva et al. 2003; Rioux et al. 2009; Pielesz and Biniaś 2010; Skriptsova et al. 2010; Stengel et al. 2011; Anastyuk et al. 2012; Mak et al. 2013). More recently, in vitro studies have provided insight into some structure-function relationships of FCPs (Cumashi et al. 2007; Ushakova et al. 2009; Ustyuzhanina et al. 2013, 2014).

It can be concluded that knowledge of the beneficial effects of algae and their extracts as food additives for humans lags far behind that on which diets have been formulated for commercially important species in aquaculture and agriculture. The number of species exhibiting benefits is wide ranging from invertebrates (nematodes, shrimp, abalone) and finfish (sea bream to salmon) to farm animals including poultry and mammals (both ruminants and monogastric species) (reviews: Craigie 2010; O’Sullivan et al. 2010; Rajauria 2015; Heuzé et al. 2016; Makkar et al. 2016). Algal-based products Tasco™ from *Ascophyllum nodosum* and Ocean Feed™ (a blend of brown, green and red macroalgae) are commercially marketed as feed additives to improve performance, stimulate immune reactions, mitigate sea lice damage in salmonids, and other benefits. Notable is the Alternative Feeds Initiative to develop alternative dietary ingredients (NOAA 2011). In addition to conventional methods of measuring animal performance, molecular techniques have been applied to buttress claims of efficacy (cf. Kulshreshtha et al. 2014; Liu et al. 2015). Bearing in mind ethical considerations, similar approaches may be adapted to facilitate the assessment of the benefits of macroalgal ingestion by humans.

## Vitamins

Vitamins are essential organic micronutrients, which an organism cannot synthesize directly in sufficient quantities and so instead must obtain from the diet. Well-known human vitamin-deficiency diseases include beriberi (deficiency in thiamine, vitamin B_1_), pellagra (niacin), pernicious anemia (cobalamin, vitamin B_12_), and scurvy (ascorbic acid, vitamin C) (Stabler and Allen 2004; Martin et al. 2011). These compounds serve as precursors for essential enzyme cofactors and are needed for essential metabolic functions (Fig. 4). Since animals have lost the capacity to synthesize these cofactors, they must obtain them from external sources. Algal foods are rich in vitamins. Several sea vegetables—laver (*Porphyra umbilicalis*), sea spaghetti (*Himanthalia elongata*), and *Gracilaria changii*—contain levels of vitamin C comparable to common vegetables such as tomatoes and lettuce (Norziah and Ching 2000; Ferraces-Casais et al. 2012), while the vitamin C content described for the brown seaweed *Eisenia arborea* (34.4 mg (100 g)^−1^ dry wt) approaches those reported for mandarin oranges (Hernandez-Carmona et al. 2009). The vitamin content of individual algal species discussed in this section, including details of sample origin and handling, is compiled in Online Resource 4.

Sea vegetables also are a good source of B-group vitamins (particularly B_1_, B_12_), as well as the lipophilic vitamin A (derived from the carotenoid β-carotene) and vitamin E (tocopherol). Kelp (*Macrocystis pyrifera*) can contain levels of α-tocopherol (the most biologically active form of vitamin E) at par with plant oils rich in this vitamin, such as palm, sunflower seed, and soybean oils (Ortiz et al. 2009; Skrovankova 2011). Moreover, values of β-carotene (pro-vitamin A) found in the seaweeds *Codium fragile* and *Gracilaria chilensis* can exceed those measured in carrots (Ortiz et al. 2009). The vitamin composition of microalgae can be equally remarkable. Fabregas and Herrero (1990) showed that *Tetraselmis suecica*, *Isochrysis galbana*, *Dunaliella tertiolecta*, and *Chlorella stigmatophora* were particularly rich in lipid-soluble (A and E) and B-group vitamins [including vitamins B_1_, B_2_ (riboflavin), B_6_ (pyridoxal), and B_12_]. The concentrations of several vitamins, including E, B_1_, and β-carotene, exceeded those in conventional foods considered to be rich sources of these compounds (Fabregas and Herrero 1990). It is clear then that algal foods can be an excellent source for a wide range of these essential micronutrients.

Even so, variability between samples can make direct comparisons among studies difficult (e.g., Chan et al. 1997; McDermid and Stuercke 2003; Hernandez-Carmona et al. 2009). Part of the variability may lie in the sample processing methods (Skrovankova 2011) as observed for other phytochemicals (Ling et al. 2015); for example, analysis of freeze-dried and oven dried samples of *Sargassum hemiphyllum* yielded substantially different vitamin C contents (Chan et al. 1997). But differences also can be due to environmental and seasonal factors. For instance, there are notable variations in the levels of β-carotene and vitamin C between samples of *Ulva fasciata* collected from different sites (McDermid and Stuercke 2003) (Online Resource 4). Monthly quantitation of vitamins C, B_2_, B_1_, and A concentrations in *Eisenia arborea* over a 12-month period revealed levels were lowest in the summer months (June, July, August) and reached the highest concentrations in April/September (for vitamins C, B_2_, B_1_) and January (for provitamin A) (Hernandez-Carmona et al. 2009). The proximate cause for these patterns is unknown, as is the effect of growth conditions on the content and composition of vitamins in algal foods, so this is an important topic for future research.

Algal foods offer one of the few vegetarian alternatives for cobalamin (vitamin B_12_) in the diet. Cobalamin is not required or synthesized by higher plants (Croft et al. 2005) so fruits and vegetables are poor sources of vitamin B_12_, which explains why vitamin B_12_-deficiency is common among people following strict vegetarian or vegan diets (Haddad et al. 1999; Waldmann et al. 2004; Allen 2008). Over half of microalgal species surveyed have a metabolic requirement for B_12_, and contain large amounts (Online Resource 4), but they cannot synthesize it (Croft et al. 2005; Helliwell et al. 2011). Cobalamin is synthesized only by prokaryotes (Warren et al. 2002), and it has been shown that B_12_-synthesizing bacteria are closely associated with or reside on eukaryotic algal surfaces (Croft et al. 2005; Wagner-Döbler et al. 2010). *Pyropia yezoensis* (nori) contains up to ∼0.06 mg vitamin B_12_ (100 g)^−1^ algal dry wt, comparable to that found in beef liver (Watanabe et al. 1999b; Takenaka et al. 2001). Lower levels are found in other sea vegetables such as kelps (including wakame) and hijiki, although reported concentrations vary among studies, possibly reflecting differences among strains, growing conditions, or harvesting periods (Watanabe et al. 1999a; Miyamoto et al. 2009). Given that the ultimate source of vitamin B_12_ is bacteria, changes in the character and magnitude of the epiphytic prokaryotic communities related to region or algal physiological state may contribute to variation in vitamin content; these factors currently are poorly quantified.

There is uncertainty about whether the magnitude of vitamin concentration in algal foods reflects their nutritional value. Dagnelie et al. (1991) investigated how sea vegetables affected the hematological status of B_12_-deficient children and concluded that the algal-derived vitamin B_12_ was not bioaccessible to humans. However, their very small treatment group (*n* = 5) may have been insufficient to draw firm conclusions. Takenaka et al. (2001) showed that feeding nori to vitamin B_12_-deficient rats yielded a 1.9-fold increase in hepatic levels of total B_12_ compared to those without nori supplementation. Similarly, increased consumption of *Chlorella* or nori by vegan participants prevented B_12_ deficiency (Rauma et al. 1995). However, there are few data on which to base quantitative estimates of the portion of algal vitamins that are absorbed during digestion.

One approach to assessing the availability of vitamins is to distinguish among their different chemical forms. The uptake of cobalamin-based compounds, referred to more broadly as corrinoids, is largely governed by the gastrointestinal absorption system rather than their chemical liberation via digestive chemical processes (Russell-Jones et al. 1999). Pseudovitamin B_12_ (or pseudocobalamin) differs from cobalamin in its lower axial ligand (Stupperich and Krautler 1988), and this affects affinity of the mammalian B_12_-binding protein intrinsic factor (IF) for the compound, thereby limiting its absorption in the intestine (Stupperich and Nexo 1991). This difference has human health implications because commercially produced vitamin B_12_ supplements derived from the cyanobacterium *Arthrospira* sp. (spirulina) consist largely of pseudovitamin B_12_ (Watanabe et al. 1999b; Watanabe 2007a), reducing their nutritional value. In contrast, “green” (*Ulva* [formerly *Enteromorpha* sp.]) and “purple” (*Pyropia* [formerly *Porphyra*] sp.) laver contain substantial amounts of biologically available B_12_ (Watanabe et al. 1999b), and indeed, rats fed purple laver improved their B_12_ status (Watanabe et al. 1999b). A recent study has established that the vast majority of cyanobacteria synthesize pseudocobalamin, whereas eukaryotic algae that are dependent on B_12_ for growth are like animals in that they require cobalamin (Helliwell et al. 2016). Thus, sea vegetables are likely to be a more reliable source of the appropriate form of this vitamin, although again this will be determined by the prokaryotic community associated with the algae.

These findings highlight the need for rigorous care in the analytical determinations of the vitamin content of algal foods. Bioassays using B_12_-dependent bacteria such as *Lactobacillus delbruekii* ssp. *lactis* (ATCC7830) are inadequate because, unlike humans, these bacteria do not discriminate between vitamin B_12_ and pseudovitamin B_12_. An alternative radioisotope dilution assay (RIDA) is likely to better reflect the functional B_12_ content (Watanabe 2007a). Distinguishing among bioavailable and non-bioavailable vitamin forms will be crucial (Watanabe 2007b). Complicating these analyses further is evidence that commercial processing methods can alter the vitamin chemistry sufficiently to affect uptake. For example, Yamada et al. (1999) showed that air-drying *Pyropia tenera* (asakusa-nori) produced B_12_ analogs that are biologically inactive. Drying by lyophilization might have better nutritional outcomes (Takenaka et al. 2001), although this has yet to be rigorously demonstrated. Other factors of particular importance to preserving vitamin content include washing methods, storage temperature, light, and moisture content (Online Resource 1, Brown 1995; Jimenez-Escrig et al. 2001; Lage-Yusty et al. 2014). There is a strong need for more detailed investigations into how the nutritional quality of sea vegetables is affected by processing methods suited for commercial-scale production.

The bioavailability of other algal-derived vitamins is also underexplored. Vitamin E encompasses eight different forms (tocopherols and tocotrienols) that differ in their biological activity (α- and γ-tocopherols are the most active). Although much less is known about their relative bioavailability compared to the vitamin B_12_ analogs, it is clear that their relative contributions affect the nutritional quality of foodstuffs (Ortiz et al. 2009). An additional concern with fat-soluble vitamins is that they must be consumed with lipid-rich foodstuffs to ensure efficient intestinal absorption (Skrovankova 2011). Although this co-dependence is understood, there currently are few data on this dependence for edible-algal species.

Most studies on algae and vitamins often focus either on analysis of vitamin concentrations in algae (e.g., Ortiz et al. 2006, 2009; Hernandez-Carmona et al. 2009; Matanjun et al. 2009; Ferraces-Casais et al. 2012) or testing the value of an algal product as a functional food (e.g., Dagnelie et al. 1991; Rauma et al. 1995; Takenaka et al. 2001), but not both. Ideally, studies combining these two approaches should be adopted to gain meaningful insights on the true quality of algal foods as vitamin sources (Takenaka et al. 2001).

Finally, there are the ecological challenges to gaining a broad picture of algal foods as a nutritional source of vitamins. Vitamin production and metabolism can vary considerably across diverse algal lineages (Croft et al. 2006; Helliwell et al. 2011, 2013). One approach that may help reveal this complexity would be a high-throughput screening of promising algal food candidates with next-generation sequencing techniques coupled with bioinformatics to search for vitamin-biosynthesis pathways. Nevertheless, there will be continued the need for careful analytical characterizations and bioavailability testing because the up- or down-regulation of gene expression almost certainly will be environmentally regulated.

## Antioxidants

It is not surprising that there is a very broad literature on marine algae as sources of antioxidant compounds for human diets. Photosynthetic energy acquisition and transformations necessarily involve continuing redox disequilibria, with the production of reactive species that can decrease lifespan and evolutionary fitness. Microalgae and macroalgae, like other life forms, contain antioxidant organic compounds and enzymes that limit this oxidative damage, which results primarily from reduced states of oxygen—the “reactive oxygen species”—including the superoxide radical anion (O_2_^−·^; O_2_ + 1e^−^), hydrogen peroxide (H_2_O_2_; O_2_ + 2e^−^), the hydroxyl free radical (HO^·^; O_2_ + 3e^−^), and singlet oxygen (^1^O_2_) (Halliwell and Gutteridge 2007). Whereas the antioxidant benefits of several terrestrial plant foods are established, much less is known about whether algal foods provide similar benefits.

The reactive oxygen metabolism in marine algae is diverse and complex, given the wide range of antioxidant compounds (Cornish and Garbary 2010), but an extension to any beneficial response from human consumption of these substrates is far less certain. Antioxidant activity can have two forms: the activity of antioxidant enzymes or the production of molecules that serve as sacrificial scavengers of reactive oxygen species. There also are two broad categories of antioxidant activity: limiting reactive oxygen species within the digestive tract, thereby decreasing oxidative stress on the gut microbiome and epithelial cells, or transport into the blood for distribution throughout the body. Evidence for direct transport is very limited, as there seems to have been no systematic study of digestive uptake of these compounds. In one study, Okada et al. (2009) examined the bioaccessibility of astaxanthin extracted from the green alga *Haematococcus* (Fig. 2) as judged from the concentration in blood serum, as a function of the timing of the ingestion of astaxanthin relative to a meal, and whether the subjects were smokers or non-smokers. Astaxanthin increased more in serum when the dose was taken 10 min after a meal rather than 2 h before, evidence of complex factors affecting its bioaccessibility. The ingestion (and topical application) of polyphenols of brown algae inhibited UVB radiation-induced skin carcinogenesis in mice (Hwang et al. 2006), and while this bioactivity remains to be determined for humans, it provides evidence that algal foods have significant functional food potential.

The foremost enzymes that restrict oxidative damage in algae and terrestrial foods include the superoxide dismutases that remove superoxide radical anions, and catalases and peroxidases, that convert hydrogen peroxide to water. Superoxide dismutases in cyanobacteria have Ni, or mixtures of Fe, Mn, and Ni, as the metal, whereas eukaryotic algae have Mn or Fe, or some combination of Fe, Mn, and Cu + Zn (Wolfe-Simon et al. 2005). Catalase has an Fe-containing heme cofactor while peroxidases use a reductant to convert hydrogen peroxide to water. Of these enzyme cofactors, Cu and Zn, and particularly Fe are used in numerous human metabolic pathways. Since the ingested antioxidant enzymes are digested in the intestine, the only effect the enzymes can have in the animal is through uptake of the metal cofactors across the intestinal epithelium. The possible effects on the intestinal microbiome of any undigested enzyme, or of the released metal cofactors, have not been investigated.

There is a stronger linkage between selenium in food and antioxidant capacity in metazoans such as mammals. Selenium is an essential metal in metazoans and some algae for the production of Se-requiring glutathione peroxidase, used to metabolize hydrogen peroxide and lipid hydroperoxides (Halliwell and Gutteridge 2007; Perez et al. 2007; Gobler et al. 2011). Analyses of the elemental contents of microalgae (Quigg et al. 2011) and macroalgae (Tuzen et al. 2009; Pereira 2011) rarely include Se, even though it is present in both (Fournier et al. 2005). Se readily bioaccumulates in algae (Cases et al. 2001; Fournier et al. 2005), and Se-deficiency in rats can be alleviated by oral supplementation with Se-rich *Arthrospira* (spirulina), as indicated by increased activity of (Se-containing) glutathione peroxidase in the kidneys and liver (Cases et al. 2001). However, increases in this enzyme activity were greater in rats supplied selenite or selenomethionine (more reactive species) than with the same dosage of Se-rich cyanobacterium, likely due to lower bioavailability of the cyanobacterial Se. The factors regulating Se content of algal foods and its availability are prime research topics for the future.

Under normal metabolic conditions, the production of hydroxyl radicals and singlet oxygen cause almost immediate damage, essentially reacting with the first oxidizable molecule that they encounter. In these cases, “sacrificial” scavengers (of HO^·^) and quenchers (of ^1^O_2_) often are the only recourse for limiting damage once the free radicals are produced (Smirnoff and Cumber 1989; Telfer et al. 1994a, b; Sunda et al. 2002; Ledford and Niyogi 2005; Halliwell and Gutteridge 2007; Ledford et al. 2007). Algae contain a wide range of molecules capable of free radical scavenging activity in vitro and in vivo. These include the water-soluble ascorbate (vitamin C) and certain compatible solutes (osmoprotectants), and the lipid-soluble α-tocopherol (vitamin E) and carotenoids such as astaxanthin (Halliwell and Gutteridge 2007). Mycosporine-like amino acids, mainly considered as UV screening compounds, are also antioxidants (Oren and Gunde-Cimerman 2007) as are a range of other solutes that act as scavengers and quenchers of reactive oxygen species in algae (Cornish and Garbary 2010). HO^·^ scavengers include glycerol (Smirnoff and Cumber 1989), mannitol (Smirnoff and Cumber 1989; Shen et al. 1997; Larson et al. 2002), L-proline (Smirnoff and Cumber 1989), dimethylsulfoniopropionate (Sunda et al. 2002), and floridoside and isofloridoside (Li et al. 2010), although glycine betaine (or betaine: trimethylglycine) does not have this property (Smirnoff and Cumber 1989; Shen et al. 1997). Given that algal osmoprotectants are necessarily present in high concentrations (≥ 0.1 mol L^−1^) in metabolically diverse compartments (cytosol, plastid stroma, and mitochondrial matrix), there is potential for them to have functional food roles. However, preliminary experiments showed that none of these compounds interact with O_2_^−·^ (Smirnoff and Cumber 1989), unlike β-carotene and other carotenoids such as fucoxanthin that quench ^1^O_2_ as well as scavenging HO^·^ and O_2_^−·^ (Halliwell and Gutteridge 2007; Sachindra et al. 2007). Other algal components that scavenge free radicals are phenolic compounds (Ragan and Globitza 1986) including halophenols (Li et al. 2011) and phlorotannins (Shibata et al. 2007) and, as noted above, alginate (Zhao et al. 2012; Zhou et al. 2012) and sulfated polysaccharides (Barahona et al. 2012).

Most studies of the bioavailability of algal antioxidant products remain at the entry level with respect to human effects: in vitro testing of extract bioactivity on cell lines. Nwosa et al. (2011) confirmed and extended previous work showing the antioxidant activities of polyphenolic extracts from four species of edible marine algae in inhibiting Caco-2 colon cancer cell proliferation and α-glucosidase activity (see below): the green alga, *Ulva lactuca*, the brown algae *Alaria esculenta* and *Ascophyllum nodosum*, and the red alga *Palmaria palmata. Ulva lactuca* had a low yield of polyphenols relative to the other algae, but the brown and red algal polyphenolic extracts performed as well as antioxidants. However, Nwosa et al. (2011) illustrated that the method of preparing the extracts from marine algae can significantly alter their antioxidant efficacy (see also Ling et al. 2015), highlighting the need for caution in comparisons of antioxidant performance among studies. With this possible caveat, most work on antioxidant activity of algal phenols has involved red algae; some bromophenols from the marine red alga *Rhodomela confervoides* have greater in vitro antioxidant activity than ascorbate (Li et al. 2011). Olsen et al. (2013) showed that bromophenols extracted from the red alga *Vertebrata lanosa* significantly inhibited oxidant effects and lipid peroxidation in cultures of human fetal lung (MTC-5) and human hepatocellular liver carcinoma (HepG2). In this case, it was shown that bromophenol can enter cells, and thus potentially can move from the gut lumen into the blood stream. Overall, there is a strong need for more work on the in vivo effects of the antioxidant properties of phenols and other algal food constituents in mammals, and humans in particular.

Instead of serving to facilitate the control of reactive oxygen species, some algal components can inhibit their production, but most studies do not adequately distinguish between the decreased production and increased removal of oxidants. For example, dietary ingestion of phycocyanin, taken up from the gut as the chromophore component phycocyanobilin, and related bile pigment metabolites inhibits the generation by NADPH oxidase of O_2_^−^, which has a key role in numerous disease syndromes, e.g., antigen expression, angioplasty, cancers, glycemia and lipidemia, hypertension, immunostimulation, and age-related maculopathy (reviewed by McCarty 2007). This industry-sponsored but balanced and authoritative review shows that *Spirulina* spp. (most now transferred to *Arthrospira*) are a prominent cyanobacterial source of phycocyanobilin, a dietary supplement worthy of in-depth study.

A class of compounds attracting increasing attention are the phlorotannins found in brown algae, which have extraordinary though inconsistent antioxidant properties (see in Wang et al. 2014), in part due to the methods of extraction (Nwosa et al. 2011). The vast bulk of this work was done in vitro, much of it studying the effects of phlorotannin on carbohydrate-hydrolyzing enzymes. Nwosa et al. (2011) found that extracts of *Ascophyllum* and *Alaria* inhibited Caco-2 colon cancer cell proliferation, α-amylase activity and, to a lesser extent, α-glucosidase activity, with mass spectrometric evidence indicating that the active principal(s) were phlorotannins. Kawamura-Konishi et al. (2012) also found that phlorotannin extracts of four species of *Sargassum* significantly inhibited the salivary enzyme α-amylase in vitro, and that a novel phlorotannin from *Sargassum patens* inhibited rat pancreatic α-glucosidase action on amylopectin. Iwai (2008) showed that oral administration of extracts inhibited lipid peroxidation in the plasma, red blood cells, liver, and kidney of KK-A^y^ mice, indicating that the antioxidant activity of phlorotannins had beneficial properties for reducing diabetic oxidative stress. Important recent work (Corona et al. 2016) investigated the effect of food grade phlorotannins from *Ascophyllum nodosum* in trials on human subjects. The work showed the in vitro gastrointestinal modification of phlorotannins, the occurrence in plasma and urine of metabolites of phlorotannins, and a significant increase in cytokine IL-8. To conclude consideration of phlorotannins, while in vitro studies on phlorotannins are valuable, more work along the lines of that of Iwai (2008) and Corona et al. (2016) is needed to understand the uptake and systemic properties of phlorotannins, and to determine whether the in vitro effects occur in vivo and relate to their antioxidant properties (Bohn et al. 2015).

There remain substantial knowledge gaps about the efficacy of antioxidant properties of macroalgal and microalgal foods at all levels, from characterization among species through effects on gut microbiota and transport across the gut lumen to their impacts on human physiology. This will be a valuable area of emerging research over the next decade.

## Inorganic elements

Processed seaweeds are widely used as mineral and metal nutritional supplements (e.g., Kay 1991), but the efficacy of these supplements is poorly quantified. Most studies suffer from serious experimental limitations, including short duration of the study, small sample size, and inadequate documentation of active ingredients. There is a comparatively small literature describing mineral contents of macroalgal and microalgal foods (Cabrita et al. 2016), and very little information about seasonal variations for naturally harvested sea vegetables.

The best evidence of the human nutritional benefits of sea vegetable inorganic elements is for iodine and iron, which can be highly enriched in marine macroalgae. Nutritional generalization about algal mineral contents is difficult because of sometimes large seasonal, geographic, and taxonomic variations in mineral contents of marine algae (e.g., Jensen 1993). For example, Indonesian green, brown, and red algae contain high levels of potassium, calcium, and sodium, but significantly lower levels of iron and zinc than reported for Japanese *Pyropia* (as *Porphyra*) *yezoensis*, *Ulva* (*Enteromorpha*) *intestinalis*, and *Sargassum* (*Hijikia*) *fusiformis* (Takeshi et al. 2005). These findings may indicate that macroalgal harvests from warm equatorial areas have lower mineral nutritional value than higher latitude regions (e.g., Cabrita et al. 2016); however, there are remarkably few data on which to assess the validity of such generalizations.

### Iodine

There is a long history linking seaweed consumption by humans and the reduced incidence of goiter and other thyroid disorders. Iodine deficiency causes hypothyroidism while excess iodine uptake can induce either hyper- or hypothyroidism (Miyai et al. 2008). Seaweeds are a good nutritional source for iodine, particularly in regions where other foods are deficient, but the iodine content of commercially available sea vegetables varies dramatically among species, the methods of preparation (many iodine compounds are water soluble), and the duration of storage (iodine may vaporize under humid conditions) (Teas et al. 2004a). Many macroalgae are washed and dried for storage. These processing steps did not significantly reduce iodine content in three common species (*Alaria esculenta*, *Palmaria palmata*, and *Ulva intestinalis*), but rehydration followed by boiling in water lowered iodine content by 14–75 % (Nitschke and Stengel 2016).

**Fig. 6:**
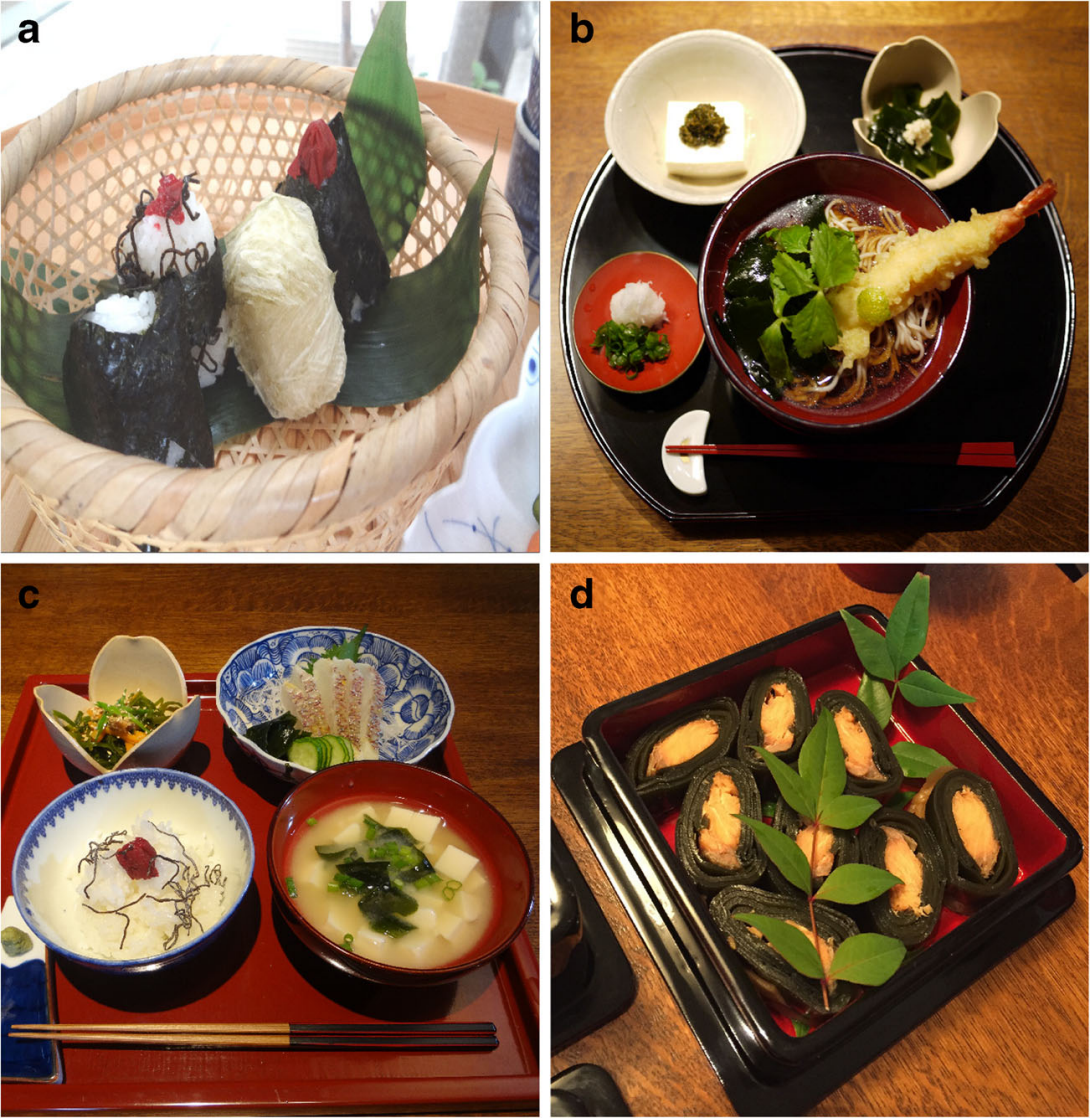
Examples of sea vegetable use in Japanese cuisine: **a** “Onigiri” is a Japanese rice ball usually wrapped by nori (*Pyropia yezoensis*) with several other ingredients: *from right to left*, rice wrapped in nori, with Japanese apricot (umeboshi) and preserved kombu (tsukudani, *Saccharina japonica* and other species of the genus *Saccharina*), wrapped with shredded kombu (torero-kombu), and wrapped in nori; **b** Tempura soba with wakame (*Undaria pinnatifida*). Wakame is used in Japan, noodles, soups, salads, pickles, and more. **c** Traditional Japanese dishes with sea vegetables: preserved kombu (tsukudani) on rice, miso soup with wakame, sliced kombu with vegetables, and sliced raw fishes (sashimi) with wakame and cucumber. **d** “Kobu-maki” is simmered food, often salmon or herring, wrapped in kombu, which is usually prepared for the New Year’s holidays. Kombu is used in several dishes and soup stock. (Courtesy of Kazuko Sato and Yoichi Sato)

Some kelps (*Laminaria* spp., *Saccharina* spp.) have high levels of iodine, and salts that include kelp powder are available commercially as a source of this vital nutrient. Not all brown algae accumulate high levels of iodine; for example, the kelps *Undaria* (wakame) and *Alaria* (“Atlantic wakame”) have lower iodine levels that are comparable to *Palmaria palmata* (dulse, a red sea vegetable) (MacArtain et al. 2007; Rhatigan 2009; Holdt and Kraan 2011; Schiener et al. 2015) (Fig. 6). In contrast, high levels of iodine in other brown macroalgae (e.g., *Laminaria*; *Saccharina*; Teas et al. 2004b; Miyai et al. 2008) have led to a strong concern that overconsumption of these particular sea vegetables can be unhealthy. Michikawa et al. (2012) reported that consumption of undefined seaweed more than 2 days per week appeared to correlate with increased risk of thyroid cancer in Japanese postmenopausal women, although Wang et al. (2016) did not find this relationship to be significant. Nevertheless, there is clear evidence that algal food consumption leads to elevated iodine levels in humans. Miyai et al. (2008) measured serum levels of thyroid hormones in conjunction with well-defined ingestion rates of kombu (*Saccharina japonica*) over short (7–10 days) and longer term (∼90 days) exposure. Urinary excretion of iodine increased significantly over the short term with increasing intake (15–30 g day^−1^ of kombu—a normal consumption for some Japanese) and suppressed thyroid function for at least 3 months. Thyroid hormones returned to normal levels when seaweed intake ceased. In this case, the absorbed iodine (20–50 mg) exceeded the recommended upper daily intake of iodine (0.2 mg day^−1^ by more than an order of magnitude; WHO 1989; Dawczynski et al. 2007; Miyai et al. 2008). Food preparation (e.g., cooking, pickling) can reduce the iodine intake, but, even then, high-iodine sea vegetables, which account for only a portion of those consumed, should be restricted in the diet (Teas et al. 2004a). Research is needed on how food preparation alters the bioavailability of iodine in different sea vegetables. Indeed, Lightowler and Davies (2002) found that established food tables (UK Department of Health, 1991) gave poor estimates of dietary iodine intake and recommended developing more reliable data on iodine in foods, including the variation within food groups.

### Iron

Macroalgae are a potentially rich source of iron for human diets. Garcia-Casal et al. (2007, 2009) measured seasonal differences in the iron content of four seaweed species common to Venezuelan waters (*Ulva* spp., *Sargassum* spp., *Porphyra* spp., and *Gracilariopsis* spp.). The *Gracilariopsis* spp. and *Sargassum* spp. had substantially higher iron content with *Porphyra* spp. having the lowest, and there was a distinct seasonal cycle whereby iron content was highest in spring and summer and lowest in fall and winter. *Sargassum*, *Ulva*, and *Porphyra* spp. have high iron content, and 15 g day^−1^ of *Sargassum* provided substantially higher amounts than daily recommended intakes (≤18 mg Fe day^−1^, Institute-of-Medicine 2001). Cabrita et al. (2016) found that Fe (and other metal) contents varied, in some cases substantially, among macroalgal species collected at the same sites and time, presumably linked to differences in metabolic requirements. Recognizing that iron consumption in macroalgae likely does not equate with uptake, the researchers quantified iron incorporation into the blood of 93 volunteers fed diets of radioactive (gamma-emitting) ^59^Fe-labeled *Ulva* spp., *Sargassum* spp., and *Porphyra* spp. (Garcia-Casal et al. 2007, 2009). Uptake of ^59^Fe was dose-dependent on sea vegetable added to maize or wheat-based meals (Garcia-Casal et al. 2007), but generally was greater than the total iron content of the different macroalgal species. Thus, in addition to enhancing total dietary iron content, the seaweeds appeared to act synergistically to facilitate iron uptake from wheat and maize, possibly related to high levels of ascorbic acid (vitamin C) that converts iron to the more readily absorbed ferrous form.

The Fe content of wild algae varies seasonally and geographically depending on the metal content of coastal waters, in addition to being species-specific (Garcia-Casal et al. 2007; Cabrita et al. 2016). Wild harvesting must then be optimized for each locale known to produce algae for optimal concentrations of inorganic nutrients. Similar constraints may apply to aquaculture crops unless they are fertilized, but there are very few data currently available on sea vegetable nutritional metal content relative to species, region, or season.

## Phytochemicals

The main non-acyl glyceride compounds in lipid membranes attracting nutritional and commercial interest are the carotenoids, especially because of their dietary importance for vision (Ben-Amotz and Levy 1996). Carotenoids, among the most widespread pigments in prokaryotic and eukaryotic photosynthetic organisms (Britton et al. 2004), function as light energy harvesters, as photoprotectants, and as antioxidants (Halliwell and Gutteridge 2007, and see below). One specific keto-carotenoid, siphonaxanthin, but not fucoxanthin, induced apoptosis in human leukemia cell lines, suggesting its potential as a chemopreventative or chemotherapeutic agent (Ganesan et al. 2011), although its efficacy remains to be determined in vivo. A subset of the carotenoids, the β-carotenes, are precursors for vitamin A (Minguez-Mosquera et al. 2008), and natural β-carotene may act as a lipophilic antioxidant in vivo, providing some protection in children exposed to radiation from the Chernobyl accident (Ben-Amotz et al. 1998).

Various strains of the green microalga *Dunaliella salina* can accumulate about 8 % of their dry weight as β-carotene, which has been marketed as a functional food (Ben-Amotz and Levy 1996; Borowitzka 2013b). The US FDA recently (2011) had no questions about a filing for the powdered form of the microalga *Dunaliella* (*bardawil*) *salina* as GRAS for use as an ingredient in food products (Agency Response Letter GRAS Notice No. GRN 000351, Borowitzka 2013b). Another main source of carotenoids is the green alga *Haematococcus pluvialis*, for which cultures have developed industrially in several countries (Cysewski and Lorenz 2004). Carotenoid supplements, including β-carotene, are effective in improving the carotenoid supply in breast milk at early lactation (Nagayama et al. 2014). However, the optimal levels of β-carotene remain controversial. Undefined β-carotene supplements increased lung cancer risk in smokers or people exposed to asbestos (Druesne-Pecollo et al. 2010), while earlier work showed no reduction in the incidence of lung cancer among male smokers after 5 to 8 years of dietary supplementation with alpha-tocopherol or β-carotene (Albanes et al. 1995).

Phenolic compounds such as flavonoids and phenolic acids serve as antioxidants. Brown macroalgae (*Alaria esculenta*, *Ascophyllum nodosum*, *F. vesiculosus*, *Saccharina latissima*) had 2–15 times the total phenolics (as mg gallic acid equivalents g^−1^ dry matter) as red species (*Chondrus crispus*, *Meristotheca papulosa*, *Palmaria palmata*, *Sarcodiotheca gaudichaudii*) (Tibbetts et al. 2016). Total phenolic content was inversely related (*r* = −0.81) to in vitro protein digestibility. Phenolic compounds are challenging to extract and characterize, but improvements in analytical methodology are expected to facilitate more detailed investigation of these antioxidants in algae. Drying *Kappaphycus alvarezii* in sunlight significantly reduces total phenolic, flavonoid, anthocyanin, and carotenoid content compared with samples dried in ovens or in the shade (Ling et al. 2015).

## Safety

Consumption of any food is not without risk, so the promotion of algal consumption must also consider potential harm to consumers. Possible risks associated with algae include excess intake of toxic metals, allergenicity, cyanotoxins, and certainly secondary metabolites (e.g., prostaglandins, kainoids) as well as contamination with pathogens, radioisotopes, and toxic synthetic compounds.

### Metal toxicity

Under normal, pristine conditions, metal uptake improves the nutritional quality of algal foods, but excessive uptake can lead to toxicity. Moreover, a substantial amount of metal associated with macroalgae can exist as colloidal-sized particles sorbed to algal surfaces (Gadd 2009; Turner et al. 2009), so surface chemistry and algal physical structures can affect metal content in addition to metabolic processes. The efficacy of macroalgae as metal collectors is why they are effective sentinel organisms for detecting anthropogenic signatures in coastal waters (Melhuus et al. 1978; Leal et al. 1997; Brown et al. 1998; Mardsen and De Wreede 2000); a feature in conflict with their use as a food source.

Despite these caveats, some general patterns appear in the literature. Metal content tends to differ among phylogenetic groups, with brown algae typically possessing higher levels of most metals in comparison with red or green algal species (Foster 1976; Munda and Hudnik 1991; Stengel and Dring 2000; Al-Masri et al. 2003; Benkdad et al. 2011). But there are exceptions. Phaneuf et al. (1999) observed that the green alga *Ulva lactuca* and *Ulva* (as *Enteromorpha*) spp. in the St. Lawrence River estuary had greater concentrations of Co, Cr, and Cu, while the brown algae *F. vesiculosus*, *Laminaria longicruris* and *Fucus distichus* had higher As and Cd concentrations (though all below regulatory levels). These species-specific differences among algae from the same environment may reflect differing metabolic affinities for these metals. Algal uptake of Cd, Cu, Co, Zn as well as Pb occurs via transporter-mediated processes, as indicated by their uptake kinetics (e.g., Garnham et al. 1992; Knauer et al. 1997; Mehta et al. 2002; Francois et al. 2007; Costa et al. 2011). As noted, however, colloidal sorption to algal surfaces can be a contributing factor, and there are few data on colloidal metal concentrations in coastal waters (Wells 2002). Regardless, macroalgae clearly can be a vector for toxic metal transfer to humans, especially if harvested from contaminated habitats.

Perez et al. (2007) studied the concentrations of many elements in *Pyropia* (as *Porphyra*) *columbina* and *Ulva* sp. in two Argentinian regions having different exposure to human activities. They found a wide range of metal content between these species with *Pyropia columbina* being a stronger accumulator of As, Cd, Mo, and Se, while *Ulva* spp. tended to accumulate more Cr, Pb, and Ni. There also were significant seasonal variations in metal loading with *Pyropia columbina* having maximum Cd concentrations during winter while *Ulva* sp. showed highest Pb concentrations during summer. This seasonality may result not only from metabolic controls but also oceanographic influences. For example, Riosmena-Rodríguez et al. (2010) found elevated concentrations of Cd in several algal species during April that appeared to be related to local upwelling events. It is unclear to what extent the presence of these metals could affect human health, as their bioavailability was not assessed.

Metal accumulation will be influenced by both the geochemical conditions and also by metabolic control as a function of ecological growth strategies or seasonally determined productivity (Stengel et al. 2004). Schiener et al. (2015) found the concentrations of major metal salts of Ca and K and the trace element Fe to more than double during spring and summer months in the brown algae *Laminaria digitata*, *Laminaria hyperborea*, and *Saccharina latissima*, but concentrations of other trace metals did not vary in a seasonal pattern. Although concentrations of Cd, Co, Cr, Cu, Fe, Hg, Mn, Ni, Pb, or Zn differed greatly among *F. vesiculosus*, *Ascophyllum nodosum*, *Laminaria longicruris*, *Palmaria palmata*, *Ulva lactuca*, and *F. distichus* collected from the St. Lawrence River estuary, Canada, all were at very low levels and there were no metal-related health risks (Phaneuf et al. 1999). Similarly, a study of the metal content of seaweeds washed up on the Brazilian coast also showed that some toxic metals were at levels considered harmful, although with remediation of nearby industrial contamination sources these macroalgae represent a potential food alternative for humans (de Oliveira et al. 2009). In one of the more comprehensive recent analyses, Dawczynski et al. (2007) measured concentrations of six trace elements (Fe, Mn, Zn, Cu, Se, and I) and four ultra-trace elements (As, Pb, Cd, and Hg) in 34 commercially available red and brown macroalgal products originating from China, Japan, and Korea. They found that for normal consumption, daily intakes of Fe, Mn, Cu, and Se are well below daily intake recommendations of the German Society of Nutrition (DGE) and the provisional tolerable weekly intake (PTWI) values of the World Health Organization (WHO), and the ultra-trace elements were present at low, harmless concentrations. Turner et al. (2008) measured the uptake of the trace metals, Pd, Cd, Hg, and Pb, by *Ulva lactuca* along the salinity gradient (S = 15–35) under well-controlled laboratory conditions. Only Cd displayed salinity-dependent uptake rates, with rates decreasing at higher salinity. Their findings also showed that the presence of environmentally relevant concentrations of dissolved humic substances (3 mg L^−1^) suppressed slightly the sorption of Pd and Hg, while moderately enhancing Pb sorption via adsorption to the algal surfaces; the uptake (internalization) of Pb was inhibited by humic substances. It is clear that processing methods that help to eliminate metals bound to algal cell walls/surfaces would be advantageous, but there is little information available on this topic.

There is thus potential for some metals to reach harmful concentrations in edible seaweeds, but there is no information on how bioaccessible or bioactive most algal metals are in human digestion. Worse, there is no consensus on a uniform or even optimal approach to quantify the bioavailability of metals. Recent advances using bio-digestive reactor approaches (e.g., Moreda-Pineiro et al. 2012) likely provide the way forward, but this remains to be established.

### Arsenic

Chronic exposure to inorganic arsenic (iAs: arsenite, As(III), arsenate, As(V)) leads to a higher incidence of several cancers including skin, lung, and urinary tract cancers. The International Agency for Research on Cancer (IARC) classifies iAs as human carcinogens (Group I), while the biological metabolites dimethylarsinic acid (DMA) and monomethylarsonic acid (MMA) are classified as possibly carcinogenic (Group 2B) in humans (IARC 2012). Toxic effects of As include disruption of oxidative phosphorylation, generation of reactive oxygen stress (ROS), enzyme inhibition, and epigenetic changes (IARC 2012; NRC 2013). Exposure to iAs occurs as humans drink water enriched in As by natural geochemical processes or poor agricultural/manufacturing/mining practices and through the diet including from foods (e.g., rice) grown in contaminated soils (NRC 2013; Zhu et al. 2014; Li et al. 2016). All seafoods contain arsenic, which enters cells through phosphate transporters and aquaglyceroporins (Bhattacharjee et al. 2008; Cooney et al. 1978; Zhao et al. 2010). The World Health Organization (WHO)’s provisional maximum level of iAs in drinking water is 10 µg L^−1^ (WHO 2016).

There are more than 50 arsenic species in seafood, but the absolute arsenic content of a seafood does not predict health risk because marine organisms have evolved detoxification strategies in which iAs is converted to methylated (organic) forms. Fish and crustaceans convert most iAs into arsenobetaine; humans excrete arsenobetaine, and it is not considered to be toxic (Francesconi 2010, Molin et al. 2015 [see their Table 1 for structures]). Algae, and mollusks that eat algae (e.g., oysters, clams), convert most iAs to arsenosugars, and there is some evidence that when phosphate levels are low, iAs may be converted to As-phospholipids that have a role in algal membrane function (Cooney et al. 1978; Garcia-Salgado et al. 2012). It is reassuring that fractionation studies show most macroalgae contain very little inorganic As in comparison to arsenosugars, although measurement of iAs in algae may be less reliable than in plants (de la Calle et al. 2012; Diaz et al. 2012; Hansen et al. 2003).

One of the major, excreted metabolites from algal arsenosugars in humans is dimethylarsinic acid (DMA), which the IARC (2012) considers to be possibly carcinogenic to humans (Group 2B) (IARC 2012). In vitro trials with human HepG2 cells showed that DMA toxicity occurred only at levels that were 400× the maximum DMA found in the urine of a human volunteer during clearance of arsenosugar (Raml et al. 2005). However, the rate of clearance of DMA and other metabolites of an arsenosugar that was experimentally ingested appears to vary in different individuals; for example, although 4-day urinary excretion removed ≥85 % of the As represented in an ingested arsenosugar in four individuals, two other individuals excreted only 4–15 %. This difference might reflect either retention of As, or perhaps that they absorbed less arsenosugar from the digestive tract (Raml et al. 2009).

Although most algae naturally synthesize arsenosugars from the iAs they take up from seawater, a few brown macroalgae contain a significant fraction of total As as iAs (*Laminaria digitata, Laminaria hyperborea*: Hansen et al. 2003, Taylor and Jackson 2016; *Sargassum* spp.: Nakamura et al. 2008, Magura et al. 2016). The sea vegetable hijiki (*Sargassum fusiforme*) contains unusually high levels of iAs (e.g., 60 µg g^−1^ dry wt hijiki, 0.4–2.8 µg g^−1^ cooked hijiki) in comparison to its arsenosugar content (Francesconi 2010; Nakamura et al. 2008; Rose et al. 2007). Nevertheless, there are at least three issues that bear upon As toxicity to humans: the chemical speciation of As in the food item, the bioaccessibility after cooking (Devesa et al. 2008; Ichikawa et al. 2006), and the metabolism of As in the individual (Choi et al. 2010; Raml et al. 2009). Ichikawa et al. (2006) reported that 88.7–91.5% (w/w) of As in hijiki was removed by cooking. Nakamura et al. (2008) determined inorganic As (III+V) extracted during a simulated gastric digestion (pepsin) from cooked hijiki donated by 14 families who also provided information on their monthly consumption of hijiki. Nakamura et al. (2008) estimated that this would result in an iAs intake of 1.1 µg kg^−1^ human body weight per week and could cause a non-negligible increase in skin cancer cases by their model. Currently, the WHO does not have a Provisional Tolerable Weekly Intake level of iAs, after withdrawing an earlier PTWI of 15 µg kg^−1^ human body weight per week (WHO 2010). More research is needed on health risks from lower dose iAs intake following consumption of the few brown algae that store iAs. The National Research Council (USA) recommends data-driven statistical approaches vs. linear extrapolation to estimate low-dose As effects from studies where higher doses are used (NRC 2013). As Nakamura et al. (2008) pointed out, calculations of excess cancer risk assume that the mechanism of carcinogenesis has no threshold dose; i.e., the incidence of cancer is linearly related to As intake in a low dose range, and this may not be the case. Inorganic As and arsenosugar content of hijiki varies geographically and with manufacturing method (Shimoda et al. 2010). Canada and the UK advise consumers to avoid eating hijiki (CFIA 2012; UK FSA 2016).

By estimating As bioaccessibility through the human GI tract using modifications of methods developed by nutritionists for estimating Fe uptake, Garcia-Sartal et al. (2012) concluded that only a fraction (7–15%) of the inorganic As and arsenosugars in cooked algae such as kombu, wakame, nori, and sea lettuce is bioaccessible. More research is needed to understand how different cooking processes, the particular algal food matrix, and the gut microbiome modify arsenosugar bioaccessibility and then better definition of the interaction of other As species produced in the body with cellular metabolites and macromolecules before their excretion (NRC 2013; Molin et al 2015; Carlin et al 2016). Experiments at all levels are important but it is particularly important to move beyond tests of toxicity using in vitro cell cultures in order to understand whether there is any risk of consuming any sea vegetable. The quantities of bioaccessible arsenic as iAs and/or arsenosugars in most sea vegetables appear too low to pose risk to individuals unless there is co-exposure to much higher levels of arsenic through high iAs-drinking water (≥10 µg L^−1^) or foods or environments contaminated by As pollutants.

### Bromine

Less well-recognized toxic effects can arise from excess intake of sea salt minerals, such as bromine, which can cause nerve, DNA, and organ damage in mammals (e.g., Boyer et al. 2002). Bromine concentrations in the urine of human female subjects in China, Japan, and Korea have been shown to correlate with their seafood intake, with macroalgae apparently being a major source (Kawai et al. 2002). However, this correlation was skewed by the consumption of terrestrial crops treated with methyl bromide, which can significantly increase total bromine intake. It is an important reminder for the need to quantify total exposure of an individual to metal intake to evaluate the effects of algal consumption.

### Allergenicity and macroalgal toxins

Relatively little has been published on the subject of possible allergenicity of algae and their products. A young man developed anaphylaxis after the first-time consumption of a spirulina tablet (Le et al. 2014), but the source of the spirulina in this tablet, or its purity, was not given. Well-known episodes of human poisoning events have occurred after consumption of wild-harvested spirulina that contained *Microcystis* and other freshwater cyanobacteria that produce neurotoxins and hepatotoxins. This emphasizes the importance of developing large-scale, controlled cultivation and daily testing of future supplement crops (e.g., Gellenbeck 2012), similar to those used now by companies that supply large quantities of GRAS-spirulina (e.g., Cyanotech, Earthrise Farms) to the food and supplement markets (Belay 2008). A dried, milled protein preparation, Whole Algalin Protein, from *Chlorella protothecoides* produced by Solazyme, Inc. was demonstrated to be unlikely to cause food allergies in nutritional studies with rats (Szabo et al. 2013).

The amino acid kainic acid, which is found in dulse and some other red algae (e.g., *Digenia simplex*), is structurally similar to glutamate, a neurotransmitter in the brain. At high doses, kainic acid is neurotoxic and used experimentally to produce disease models in mice and other animals. Concentrations of kainic acid that damage neurons are much higher than those consumed by eating dulse, but Mouritsen et al. (2013) report that no human safety standard has been established. This is both a dose and a bioavailability issue, and it needs more study because a few dwarf individuals of *Palmaria palmata* had high levels of kainic acid (>10 mg g^-1^ dry wt, Ramsey et al. 1994), even though it was undetectable or at very low level in most Atlantic dulse (Ramsey et al. 1994; Higa and Kuniyoshi 2000; Mouritsen et al. 2013). Another amino acid in the kainoid family is domoic acid (DA), which is also found at low levels in some red algae (e.g., *Chondria armata*, in the same Rhodomelaceae as *Digenia simplex*, the first discovered kainic acid-producing red alga). DA is a strong health risk during blooms of a few diatoms (e.g., *Pseudo-nitzschia*) that are bioaccumulated by filter-feeding mollusks and become a human health risk, but DA poisoning from red algae is unknown (Higa and Kuniyoshi 2000). Traditionally in both Europe and Asia, worms were eliminated from humans and animals with red algae that contain KA or DA (Mouritsen 2013).

Many *Gracilaria* (Rhodophyta) and *Caulerpa* (Chlorophyta) species are eaten as sea vegetables, especially in the western Pacific (de Gaillande et al. 2016), but illness and some deaths have occurred when a few toxic species of these genera were sold or collected by mistake (Higa and Kuniyoshi 2000; Cheney 2016). Toxic prostaglandins (PGE_2_) are found in the Asian *Gracilaria vermiculophylla*, which invaded North American and European shores in recent decades (Hammann et al. 2016). Wounding of *G. vermiculophylla* increases synthesis of PGE_2_ from arachidonic acid within minutes (Noguchi et al. 1994; Nylund et al. 2011). Hammann et al. (2016) showed that this effect was enhanced in invasive populations, finding that this appears to be a defense against naïve herbivores in the non-native habitat. Their study characterized many additional metabolites present in *G. vermiculophylla*. Fatalities have resulted from consumption of fresh *Gracilaria edulis* (*Polycavernosa tsudai* in Navarro et al. 2015), due to toxic polycavernosides (Yotsu-Yamashita et al. 2007). Navarro et al. (2015)’s recent analysis suggests, however, that the polycavernoside is produced by filamentous cyanobacteria (*Okeania* sp.) that are sometimes associated with the *Gracilaria*.

## Summary

The considerable breadth and depth of literature on algae as nutritional and functional foods frustrates attempts for a fully comprehensive assessment of the field. It is clear that there is substantial evidence for algae as nutritional and functional foods, yet there remain considerable challenges in quantifying these benefits, and in assessing potential adverse effects. The limits to our understanding fall broadly into three areas. First is the variation of nutritional and functional composition of algae across species, seasons, and different coastal environments. The scant evidence to date suggests this variability can be substantial but it is only possible to speculate about the scope of this inconsistency. That is true also for quantification of toxic, or potentially harmful constituents present within, or adsorbed to algae. Assessments should also consider the effect of processing methods, which can increase or decrease the nutritional quality. Of the challenges ahead, these issues are the most tractable to address, given that the analytical methods are well developed.

The second, and perhaps most pressing limitation, is quantifying the bioavailability, or fraction of nutritional or functional components that actually have effect in relation to their residence time in the digestive system. These effects can manifest via translocation across the small intestinal epithelial cells into the blood, by direct interactions with the digestive epithelia, by altering uptake of other substances, by regulating the microbial consortium, or by direct contact with colonic epithelial cells in the large intestine (e.g., colon cancer). There is an increasing literature on digestive “reactor” analytical methods, and much more effort in this direction would be beneficial, but it must be recognized that bioaccessibility will be a complex function of the chemistry of the substance, the processing methods used to prepare the alga as food, the specific algal matrix containing it, the consortium of bacteria and their enzymatic competency, and the presence of other foods that may interfere or enhance uptake (or within-gut effects). Also necessary is more rigor in semantics; bioavailable, bioactive, activity, digestible, gastrointestinal absorption, and utilization are not equivalent terms but are used interchangeably in the literature. Advances in understanding bioavailability of foods in general will continue piecemeal until analytical methods and studies encompassing all of these factors become routine.

The third limitation lies in understanding how algal nutritional, and particularly functional, constituents interact in human metabolism and intermediary metabolic processes. Most investigators studying these questions report in vitro experiments or in vivo experiments employing direct introduction of purified algal constituents. These methods are useful probes to identify and mechanistically understand *potential* effects of the consumption of algal-based supplements, but they are inadequate to assess nutritional and functional *foods*. Their shortcomings most often include unrealistic or uncertain doses relative to normal algal consumption, a focus on a single or narrow range of metabolic responses, and inability to assess concurrent, or synergistic effects arising from co-occurring constituents during digestion. Coupling in vivo studies with more holistic digestive research will provide for a better assessment of algae as nutritional and functional food sources.

We envisage a rare opportunity to develop a rich and rewarding collaboration among phycological, nutritional, medical, analytical, and industrial groups investigating algae as nutritional and functional foods. Part of the challenge ahead for algal scientists is understanding the complexity of merging of basic research through clinical trials and regulatory requirements to create marine food products. The recent comprehensive reviews by Finley et al. (2014) and Borowitzka (2013b) help to clarify this pathway. But the most dramatic advances will require a rethinking of experimental and collaborative approaches, and the impetus for this research will only increase as human pressures on the climate system lead us to turn more to the oceans for food that we can harvest and grow sustainably.

## Electronic supplementary material

Below is the link to the electronic supplementary material.

ESM 1 (DOCX 52.4 kb)
