# Supplementary material for: Algae as nutritional and functional food sources: revisiting our understanding
Source: J Appl Phycol. 2016 Nov 21;29(2):949–82. doi: 10.1007/s10811-016-0974-5 (PMC5387034; doi:10.1007/s10811-016-0974-5)
Supplement: Supplementary file 1 — (DOCX 52.4 kb) [file 10811_2016_974_MOESM1_ESM.docx]

Online Resource 1. The variation in protein content among selected algae.

|  | Protein Content (% dry wt) | References |
| --- | --- | --- |
| **Cyanobacteria** |  |  |
| Spirulina (*Arthrospira platensis*) | 60-70 | Ciferri (1983); Jassby (1988) |
| **Chlorophyta** |  |  |
| *Chlorella* spp. | 40-70 | Lubitz , 1962; Szabo et al., 2013; Safi et al., 2014 |
| *Ulva* spp. | 7-44 | Holdt & Kraan (2011); Pereira (2011);  Shuuluka et al. (2013) |
| **Ochrophyta: Phaeophyceae** | 3-26 |  |
| *Alaria esculenta* | 9-20 | Pereira (2011); Holdt & Kraan (2011) ; Scheiner et al., (2015) |
| *Himanthalia elongata* | 5-15 | Pereira (2011) |
| *Saccharina latissima* | 6-26 | Holdt & Kraan (2011); Pereira (2011); Scheiner et al., (2015) |
| *Undaria pinnatifida* | 12-23 | Taboada et al. (2013) |
| **Rhodophyta** |  |  |
| *Gracilaria tikvahiae* | 10.7-19.6 | Johnson et al., 2014 |
| *Palmaria palmata* (dulse) | 9.7-25.5 | Galland-Irmouli et al. (1999) |
| *Porphyra* spp. (laver) | 29-39 | Holdt & Kraan (2011); Pereira (2011);  Taboada et al. (2013) |
| *Porphyridium* | 56 | Safi et al. (2013) |
| *Pyropia spp.* (nori) | 28-44 | Holdt & Kraan (2011); Pereira (2011) |

Online Resource 2. Selected comparisons between amino acid content (g/100 g protein) in each whole food or supplement.

|  | Egg^1^ | *Arthrospira*  *platensis*  (spirulina)^1^ | *Chlorella*  *vulgaris^,2^* | *Palmaria*  *Palmata*^3^  June 1996 | *Palmaria palmata*^3^  November  1996 |
| --- | --- | --- | --- | --- | --- |
| ***Essential Amino Acids***^1^ |  |  |  |  |  |
| ***histidine*** | 2.40 | 0.90 | 1.16 | 0.0 | 1.1 |
| ***isoleucine*** | 6.60 | 0.12 | 0.09 | 0.0 | 4.1 |
| ***leucine*** | 7.00 | 7.02 | 6.91 | 0.0 | 8.3 |
| ***lysine*** | 5.30 | 5.10 | 6.30 | 0.0 | 5.5 |
| ***methionine*** | 3.20 | 1.72 | 0.60 | 3.4 | 2.7 |
| ***phenylalanine*** | 5.80 | 4.82 | 5.36 | 3.7 | 6.8 |
| ***threonine*** | 5.00 | 6.16 | 5.62 | 0.0 | 0.0 |
| ***tryptophan*** | 1.70 | 1.22 | 2.04 | 4.5 | 6.4 |
| ***valine*** | 7.20 | 2.86 | 2.85 | 10.2 | 7.0 |
| Non-essential Amino Acids |  |  |  |  |  |
| alanine | n.d. | 9.91 | 10.05 | 13.3 | 0.0 |
| arginine | 6.20 | 7.69 | 6.81 | 0.0 | 6.9 |
| aspartic acid | 11.0 | 11.82 | 10.09 | 19.8 | 27.9 |
| cysteine | 2.30 | 0.18 | 0.18 | 0 | 0 |
| glycine | 4.20 | 7.76 | 7.93 | 8.4 | 18.6 |
| glutamic acid | 12.60 | 10.50 | 8.37 | 19.8 | 0.0 |
| proline | 4.20 | 1.95 | 2.74 | 0.0 | 0.0 |
| serine | 6.90 | 6.85 | 7.17 | 12.7 | 0.0 |
| tyrosine | 4.20 | 4.83 | 7.78 | n.d. | n.d. |

^1^ Safi et al. (2014)

^2^Safi et al. (2013). Replicates of 3 independent experiments and listed ± small SDs in their Table 1.

^3^ based on Galland-Irmouli et al. (1999): see their Table 1 for monthly representations (10 of 12 mo) and the annual means ± SD.

Online Resources 3. Total amino acid composition of selected sea vegetables (mg amino acids/g dry wt of product) from independent samples (n= 5) of dried product obtained commercially by Maehre et al. (2016) from Fremtidens Mat (Oslo, Norway). (modified from Table 3 in Maehre et al., 2016). Tryptophan (an EAA) was not reported in this work.

|  | *Alaria* (raw, dried product) | *Alaria*  (boiled 30 min) | *Palmaria*  (raw, dried product) | *Palmaria*  (boiled 30 min) |
| --- | --- | --- | --- | --- |
| ***Essential Amino Acids***^1^ |  |  |  |  |
| ***histidine*** | 2.8 ± 0.4 | 3.1 ± 0.6 | 2.3 ± 0.2 | 6.2 ± 0.8 |
| ***isoleucine*** | 4.2 ± 0.6 | 4.9 ± 1.1 | 5.1 ± 0.9 | 11.3 ± 2.1 |
| ***leucine*** | 8.1 ± 1.2 | 9.6 ± 0.9 | 9.6 ± 1.2 | 21.8 ± 2.5 |
| ***lysine*** | 9.2 ± 1.1 | 10.6 ± 1.6 | 10.4±0.8 | 22.9 ± 1.7 |
| ***methionine*** | 2.6 ± 0.4 | 3.0 ± 0.9 | 2.8 ± 0.4 | 6.4 ± 0.6 |
| ***phenylalanine*** | 5.2 ± 0.3 | 5.3 ± 0.9 | 5.9 ± 0.6 | 13.6 ± 1.7 |
| ***threonine*** | 5.3 ± 0.7 | 5.9 ± 0.7 | 6.0 ± 0.7 | 12.6 ± 1.9 |
| ***tryptophan*** | - | - | - | - |
| ***valine*** | 5.9 ± 0.4 | 6.8 ± 1.4 | 7.8 ± 0.8 | 17.5 ± 2.5 |
| Non-essential Amino Acids |  |  |  |  |
| alanine | 15.5 ± 3.2 | 12.3 ± 1.6 | 12.5 ± 1.2 | 25.5 ± 2.4 |
| arginine | 6.4 ± 0.5 | 7.5 ± 0.6 | 10.4 ± 1.1 | 24.8 ± 1.9 |
| aspartic acid | 7.3 ± 1.1 | 7.7 ± 1.0 | 10.3 ± 1.0 | 18.4 ± 1.2 |
| cysteine | 0.2 ± 0.0 | 1.2 ± 1.4 | 0.7 ± 0.2 | 3.4 ± 0.3 |
| glycine | 6.5 ± 0.7 | 7.2 ± 0.7 | 8.8 ± 0.6 | 18.4 ± 1.4 |
| glutamic acid | 14.6 ± 1.7 | 14.0 ± 1.4 | 17.8± 1.2 | 30.0 ± 2.7 |
| proline | 4.2 ± 2.0 | 4.5 ± 2.7 | 7.2 ± 2.7 | 9.6 ± 2.2 |
| serine | 5.2 ± 0.8 | 5.9 ± 0.8 | 7.3 ± 0.9 | 16.7 ± 1.4 |
| tyrosine | 3.0 ± 0.5 | 4.5 ± 1.5 | 4.9 ± 0.6 | 12.4 ± 0.7 |

**Online Resource 4.** A table summarising the vitamin content of individual algal species compiled from primary studies discussed in this review. Details of sample origin, axenity and handling are given. Where possible values have been converted to be expressed as mg/100 g dry weight (dw) for ease of comparison. For values of vitamin E with the exception of Ortiz et al. (2009) it was unclear whether measurements reflect total tocopherols or specific tocopherol vitamers. Unless otherwise stated therefore we have categorised these values under the heading ‘Vitamin E’ as in the original studies. For Vitamin A, where contents were provided in International Units (IU) we took 1 IU to be biologically equivalent 0.6 µg β-carotene (Fabregas and Herrero (1990)). Blank values (‘-‘) indicate when vitamins were not detected at the method detection limit (McDermid and Stuercke (2003)). Key: * represents mean value over 10 months (March-December).

| **Species** | **Vitamin** | **Content** | **Units** | **Sample origin** | **Axenic?** | **Sample handling** | **Study reference** |
| --- | --- | --- | --- | --- | --- | --- | --- |
| *Eisenia arborea J.E. Areschoug* | **Vitamin B1** | 0.097* | mg/100 g (dw) | Natural sample (fresh), Baja California, Mexico | N | Sun-dried, ground | Hernández-Carmona et al. (2009) |
| *Tetraselmis suecica* |  | 3.23 | mg/100 g (dw) | Laboratory culture | Unknown | Oven-dried | Fabregas and Herrero (1990) |
| *Isochrysis galbana* |  | 1.4 | mg/100 g (dw) | Laboratory culture | Unknown | Oven-dried | Fabregas and Herrero (1990) |
| *Dunaliella teriolecta* |  | 2.9 | mg/100 g (dw) | Laboratory culture | Unknown | Oven-dried | Fabregas and Herrero (1990) |
| *Chlorella stigmatophora* |  | 1.46 | mg/100 g (dw) | Laboratory culture | Unknown | Oven-dried | Fabregas and Herrero (1990) |
| *Caulerpa llentillifera* |  | - | mg/100 g (dw) | Natural sample (fresh). Hilton Waikaloa, Hawai’i | N | Washed and dried | McDermid and Stuercke (2003) |
| *Codium reediae* |  | - | mg/100 g (dw) | Natural sample (fresh). RHSF, Hawai’i | N | Washed and dried | McDermid and Stuercke (2003) |
| *Enteromorpha flexuosa* |  | - | mg/100 g (dw) | Natural sample (fresh). North Shore, O’ahu | N | Washed and dried | McDermid and Stuercke (2003) |
| *Monostroma oxyspermum* |  | - | mg/100 g (dw) | Natural sample (fresh). Leleiwi, Hawai’i | N | Washed and dried | McDermid and Stuercke (2003) |
| *Ulva fasciata* |  | - | mg/100 g (dw) | Natural sample (fresh). North Shore, O’ahu | N | Washed and dried | McDermid and Stuercke (2003) |
| *Ulva fasciata* |  | - | mg/100 g (dw) | Natural sample (fresh), Ma’alaea Bay, Maui | N | Washed and dried | McDermid and Stuercke (2003) |
| *Sargassum echinocarpum* |  | - | mg/100 g (dw) | Natural sample (fresh), Onekahakaha, Hawai’i | N | Washed and dried | McDermid and Stuercke (2003) |
| *Sargassum obtusifolium* |  | 6 | mg/100 g (dw) | Natural sample (fresh), Onekahakaha, Hawai’i | N | Washed and dried | McDermid and Stuercke (2003) |
| *Ahnfeltiopsis concinna* |  | - | mg/100 g (dw) | Natural sample (fresh), Onekahakaha, Hawai’i | N | Washed and dried | McDermid and Stuercke (2003) |
| *Ahnfeltiopsis concinna* |  | - | mg/100 g (dw) | Natural sample (fresh), Kona, Hawai’i | N | Washed and dried | McDermid and Stuercke (2003) |
| *Chondrus ocellatus* |  | 9 | mg/100 g (dw) | Natural sample (fresh), Leleiwi, Hawai’i | N | Washed and dried | McDermid and Stuercke (2003) |
| *Eucheuma denticulatum* |  | - | mg/100 g (dw) | Natural sample (fresh), K¯ane’ohe Bay, O’ahu | N | Washed and dried | McDermid and Stuercke (2003) |
| *Gracilaria coropifolia* |  | - | mg/100 g (dw) | Natural sample (fresh), RHSF, Hawai’i | N | Washed and dried | McDermid and Stuercke (2003) |
| *Gracilaria parvispora* |  | - | mg/100 g (dw) | Natural sample (fresh), KKHH, Moloka‘i | N | Washed and dried | McDermid and Stuercke (2003) |
| *Porphyra vietnamensis* |  | - | mg/100 g (dw) | Natural sample (fresh), Onekahakaha, Hawai’i | N | Washed and dried | McDermid and Stuercke (2003) |
| *Eisenia arborea J.E. Areschoug* | **Vitamin B2** | 0.816* | mg/100 g (dw) | Natural sample (fresh), Baja California Peninsula, Mexico | N | Sun-dried, ground | Hernández-Carmona et al. (2009) |
| *Tetraselmis suecica* |  | 1.91 | mg/100 g (dw) | Laboratory culture | Unknown | Oven-dried | Fabregas and Herrero (1990) |
| *Isochrysis galbana* |  | 3 | mg/100 g (dw) | Laboratory culture | Unknown | Oven-dried | Fabregas and Herrero (1990) |
| *Dunaliella teriolecta* |  | 3.12 | mg/100 g (dw) | Laboratory culture | Unknown | Oven-dried | Fabregas and Herrero (1990) |
| *Chlorella stigmatophora* |  | 1.96 | mg/100 g (dw) | Laboratory culture | Unknown | Oven-dried | Fabregas and Herrero (1990) |
| *Caulerpa llentillifera* |  | - | mg/100g (dw) | Natural sample (fresh). Hilton Waikaloa, Hawai’i | N | Washed and dried | McDermid and Stuercke (2003) |
| *Codium reediae* |  | 0.4 | mg/100g (dw) | Natural sample (fresh). RHSF, Hawai’i | N | Washed and dried | McDermid and Stuercke (2003) |
| *Enteromorpha flexuosa* |  | - | mg/100g (dw) | Natural sample (fresh). North Shore, O’ahu | N | Washed and dried | McDermid and Stuercke (2003) |
| *Monostroma oxyspermum* |  | - | mg/100g (dw) | Natural sample (fresh). Leleiwi, Hawai’i | N | Washed and dried | McDermid and Stuercke (2003) |
| *Ulva fasciata* |  | 1 | mg/100g (dw) | Natural sample (fresh). North Shore, O’ahu | N | Washed and dried | McDermid and Stuercke (2003) |
| *Ulva fasciata* |  | - | mg/100g (dw) | Natural sample (fresh), Ma’alaea Bay, Maui | N | Washed and dried | McDermid and Stuercke (2003) |
| *Sargassum echinocarpum* |  | - | mg/100g (dw) | Natural sample (fresh), Onekahakaha, Hawai’i | N | Washed and dried | McDermid and Stuercke (2003) |
| *Sargassum obtusifolium* |  | - | mg/100g (dw) | Natural sample (fresh), Onekahakaha, Hawai’i | N | Washed and dried | McDermid and Stuercke (2003) |
| *Ahnfeltiopsis concinna* |  | - | mg/100g (dw) | Natural sample (fresh), Onekahakaha, Hawai’i | N | Washed and dried | McDermid and Stuercke (2003) |
| *Ahnfeltiopsis concinna* |  | - | mg/100g (dw) | Natural sample (fresh), Kona, Hawai’i | N | Washed and dried | McDermid and Stuercke (2003) |
| *Chondrus ocellatus* |  | - | mg/100g (dw) | Natural sample (fresh), Leleiwi, Hawai’i | N | Washed and dried | McDermid and Stuercke (2003) |
| *Eucheuma denticulatum* |  | - | mg/100g (dw) | Natural sample (fresh), K¯ane’ohe Bay, O’ahu | N | Washed and dried | McDermid and Stuercke (2003) |
| *Gracilaria coropifolia* |  | - | mg/100g (dw) | Natural sample (fresh), RHSF, Hawai’i | N | Washed and dried | McDermid and Stuercke (2003) |
| *Gracilaria parvispora* |  | 0.6 | mg/100g (dw) | Natural sample (fresh), KKHH, Moloka‘i | N | Washed and dried | McDermid and Stuercke (2003) |
| *Porphyra vietnamensis* |  | - | mg/100g (dw) | Natural sample (fresh), Onekahakaha, Hawai’i | N | Washed and dried | McDermid and Stuercke (2003) |
| *Caulerpa llentillifera* | **Vitamin B3** | 200 | mg/100g (dw) | Natural sample (fresh). Hilton Waikaloa, Hawai’i | N | Washed and dried | McDermid and Stuercke (2003) |
| *Codium reediae* |  | - | mg/100g (dw) | Natural sample (fresh). RHSF, Hawai’i | N | Washed and dried | McDermid and Stuercke (2003) |
| *Enteromorpha flexuosa* |  | - | mg/100g (dw) | Natural sample (fresh). North Shore, O’ahu | N | Washed and dried | McDermid and Stuercke (2003) |
| *Monostroma oxyspermum* |  | - | mg/100g (dw) | Natural sample (fresh). Leleiwi, Hawai’i | N | Washed and dried | McDermid and Stuercke (2003) |
| *Ulva fasciata* |  | - | mg/100g (dw) | Natural sample (fresh). North Shore, O’ahu | N | Washed and dried | McDermid and Stuercke (2003) |
| *Ulva fasciata* |  | 66 | mg/100g (dw) | Natural sample (fresh), Ma’alaea Bay, Maui | N | Washed and dried | McDermid and Stuercke (2003) |
| *Sargassum echinocarpum* |  | 9 | mg/100g (dw) | Natural sample (fresh), Onekahakaha, Hawai’i | N | Washed and dried | McDermid and Stuercke (2003) |
| *Sargassum obtusifolium* |  | - | mg/100g (dw) | Natural sample (fresh), Onekahakaha, Hawai’i | N | Washed and dried | McDermid and Stuercke (2003) |
| *Ahnfeltiopsis concinna* |  | - | mg/100g (dw) | Natural sample (fresh), Onekahakaha, Hawai’i | N | Washed and dried | McDermid and Stuercke (2003) |
| *Ahnfeltiopsis concinna* |  | - | mg/100g (dw) | Natural sample (fresh), Kona, Hawai’i | N | Washed and dried | McDermid and Stuercke (2003) |
| *Chondrus ocellatus* |  | 6 | mg/100g (dw) | Natural sample (fresh), Leleiwi, Hawai’i | N | Washed and dried | McDermid and Stuercke (2003) |
| *Eucheuma denticulatum* |  | - | mg/100g (dw) | Natural sample (fresh), K¯ane’ohe Bay, O’ahu | N | Washed and dried | McDermid and Stuercke (2003) |
| *Gracilaria coropifolia* |  | 70 | mg/100g (dw) | Natural sample (fresh), RHSF, Hawai’i | N | Washed and dried | McDermid and Stuercke (2003) |
| *Gracilaria parvispora* |  | - | mg/100g (dw) | Natural sample (fresh), KKHH, Moloka‘i | N | Washed and dried | McDermid and Stuercke (2003) |
| *Porphyra vietnamensis* |  | - | mg/100g (dw) | Natural sample (fresh), Onekahakaha, Hawai’i | N | Washed and dried | McDermid and Stuercke (2003) |
| *Tetraselmis suecica* | **Vitamin B5** | 3.77 | mg/100 g (dw) | Laboratory culture | Unknown | Oven-dried | Fabregas and Herrero (1990) |
| *Isochrysis galbana* |  | 0.91 | mg/100 g (dw) | Laboratory culture | Unknown | Oven-dried | Fabregas and Herrero (1990) |
| *Dunaliella teriolecta* |  | 1.32 | mg/100 g (dw) | Laboratory culture | Unknown | Oven-dried | Fabregas and Herrero (1990) |
| *Chlorella stigmatophora* |  | 2.14 | mg/100 g (dw) | Laboratory culture | Unknown | Oven-dried | Fabregas and Herrero (1990) |
| *Tetraselmis suecica* | **Vitamin B6** | 0.28 | mg/100 g (dw) | Laboratory culture | Unknown | Oven-dried | Fabregas and Herrero (1990) |
| *Isochrysis galbana* |  | 0.18 | mg/100 g (dw) | Laboratory culture | Unknown | Oven-dried | Fabregas and Herrero (1990) |
| *Dunaliella teriolecta* |  | 0.22 | mg/100 g (dw) | Laboratory culture | Unknown | Oven-dried | Fabregas and Herrero (1990) |
| *Chlorella stigmatophora* |  | 0.19 | mg/100 g (dw) | Laboratory culture | Unknown | Oven-dried | Fabregas and Herrero (1990) |
| *Tetraselmis suecica* | **Vitamin B7** | 0.08 | mg/100 g (dw) | Laboratory culture | Unknown | Oven-dried | Fabregas and Herrero (1990) |
| *Isochrysis galbana* |  | 0.1 | mg/100 g (dw) | Laboratory culture | Unknown | Oven-dried | Fabregas and Herrero (1990) |
| *Dunaliella teriolecta* |  | 0.09 | mg/100 g (dw) | Laboratory culture | Unknown | Oven-dried | Fabregas and Herrero (1990) |
| *Chlorella stigmatophora* |  | 0.11 | mg/100 g (dw) | Laboratory culture | Unknown | Oven-dried | Fabregas and Herrero (1990) |
| *Tetraselmis suecica* | **Vitamin B12** | 0.05 | mg/100 g (dw) | Laboratory culture | Unknown | Oven-dried | Fabregas and Herrero (1990) |
| *Isochrysis galbana* |  | 0.06 | mg/100 g (dw) | Laboratory culture | Unknown | Oven-dried | Fabregas and Herrero (1990) |
| *Dunaliella teriolecta* |  | 0.07 | mg/100 g (dw) | Laboratory culture | Unknown | Oven-dried | Fabregas and Herrero (1990) |
| *Chlorella stigmatophora* |  | 0.06 | mg/100 g (dw) | Laboratory culture | Unknown | Oven-dried | Fabregas and Herrero (1990) |
| *Porphyra sp.* |  | 0.0692 ± 0.0022 | mg/100 g (dw) | Local market in Kochi-city, Japan | N | Dried and ground (chemiluminescence assay) | Watanebe et al. (1999b) |
| *Porphyra yezoensis* |  | 0.0586 | mg/100 g (dw) | Fisherman's Association of Ishinoura in Akashi city, Hyogo prefecture, Japan | N | Dried , ground (chemiluminescence assay) | Takenaka et al. (2001) |
| *Enteromorpha sp.* |  | 0.025 ± 0.00054 | mg/100 g (dw) | Local market in Kochi-city, Japan | N | Dried , ground (chemiluminescence assay) | Watanebe et al. (1999b) |
| *Gracilaria changgi* | **Vitamin C** | 28.5 | mg/100 g (dw) | Natural sample (fresh), Culture Ponds, Fishery Research Institute, Kedah, (Malaysia) | N | Fresh | Norziah and Ching (2000) |
| *Laminaria spp.* |  | 1.34±0.06 | mg/100 g (dw) | Natural sample (fresh), Atlantic coast, Galicia (Spain) | N | Ground, homogenized | Ferraces-Casais et al. (2002) |
| *Porphyra umbilicalis* |  | 33.29±8.46 | mg/100 g (dw) | Natural sample (fresh), Atlantic coast, Galicia (Spain) | N | Ground, homogenized | Ferraces-Casais et al. (2002) |
| *Himanthalia elongata* |  | 46.66±5.94 | mg/100 g (dw) | Natural sample (fresh), Atlantic coast, Galicia (Spain) | N | Ground, homogenized | Ferraces-Casais et al. (2002) |
| *Palmaria palmata* |  | 0.61±0.02 | mg/100 g (dw) | Natural sample (fresh), Atlantic coast, Galicia (Spain) | N | Ground, homogenized | Ferraces-Casais et al. (2002) |
| *Eisenia arborea J.E. Areschoug* |  | 34.4* | mg/100 g (dw) | Natural sample (fresh), Baja California Peninsula, Mexico | N | Sun-dried, ground | Hernández-Carmona et al. (2009) |
| *Sargassum hemiphyllum* |  | 153 ±12.0 | mg/100 g (dw) | Natural sample (fresh), Tung Ping Chau, NE Hong Kong | N | Freeze-dried, ground | (Chan et al. 1997) |
| *Sargassum hemiphyllum* |  | 97.7 ± 12.1 | mg/100 g (dw) | Natural sample (fresh), Tung Ping Chau, NE Hong Kong | N | Oven-dried, ground | (Chan et al. 1997) |
| *Sargassum hemiphyllum* |  | 51.9 ±3.47 | mg/100 g (dw) | Natural sample (fresh), Tung Ping Chau, NE Hong Kong | N | Sun-dried, ground | (Chan et al. 1997) |
| *Tetraselmis suecica* |  | 19.1 | mg/100 g (dw) | Laboratory culture | Unknown | Oven-dried | Fabregas and Herrero (1990) |
| *Isochrysis galbana* |  | 11.9 | mg/100 g (dw) | Laboratory culture | Unknown | Oven-dried | Fabregas and Herrero (1990) |
| *Dunaliella teriolecta* |  | 16.32 | mg/100 g (dw) | Laboratory culture | Unknown | Oven-dried | Fabregas and Herrero (1990) |
| *Chlorella stigmatophora* |  | 10.02 | mg/100 g (dw) | Laboratory culture | Unknown | Oven-dried | Fabregas and Herrero (1990) |
| *Codium reediae* |  | - | mg/100g (dw) | Natural sample (fresh). RHSF, Hawai’i | N | Washed and dried | McDermid and Stuercke (2003) |
| *Enteromorpha flexuosa* |  | 300 | mg/100g (dw) | Natural sample (fresh). North Shore, O’ahu | N | Washed and dried | McDermid and Stuercke (2003) |
| *Monostroma oxyspermum* |  | 130 | mg/100g (dw) | Natural sample (fresh). Leleiwi, Hawai’i | N | Washed and dried | McDermid and Stuercke (2003) |
| *Ulva fasciata* |  | 220 | mg/100g (dw) | Natural sample (fresh). North Shore, O’ahu | N | Washed and dried | McDermid and Stuercke (2003) |
| *Ulva fasciata* |  | - | mg/100g (dw) | Natural sample (fresh), Ma’alaea Bay, Maui | N | Washed and dried | McDermid and Stuercke (2003) |
| *Sargassum echinocarpum* |  | - | mg/100g (dw) | Natural sample (fresh), Onekahakaha, Hawai’i | N | Washed and dried | McDermid and Stuercke (2003) |
| *Sargassum obtusifolium* |  | - | mg/100g (dw) | Natural sample (fresh), Onekahakaha, Hawai’i | N | Washed and dried | McDermid and Stuercke (2003) |
| *Ahnfeltiopsis concinna* |  | - | mg/100g (dw) | Natural sample (fresh), Onekahakaha, Hawai’i | N | Washed and dried | McDermid and Stuercke (2003) |
| *Ahnfeltiopsis concinna* |  | - | mg/100g (dw) | Natural sample (fresh), Kona, Hawai’i | N | Washed and dried | McDermid and Stuercke (2003) |
| *Chondrus ocellatus* |  | 0 | mg/100g (dw) | Natural sample (fresh), Leleiwi, Hawai’i | N | Washed and dried | McDermid and Stuercke (2003) |
| *Eucheuma denticulatum* |  | 200 | mg/100g (dw) | Natural sample (fresh), K¯ane’ohe Bay, O’ahu | N | Washed and dried | McDermid and Stuercke (2003) |
| *Gracilaria coropifolia* |  | - | mg/100g (dw) | Natural sample (fresh), RHSF, Hawai’i | N | Washed and dried | McDermid and Stuercke (2003) |
| *Gracilaria parvispora* |  | - | mg/100g (dw) | Natural sample (fresh), KKHH, Moloka‘i | N | Washed and dried | McDermid and Stuercke (2003) |
| *Porphyra vietnamensis* |  | - | mg/100g (dw) | Natural sample (fresh), Onekahakaha, Hawai’i | N | Washed and dried | McDermid and Stuercke (2003) |
| *Laminaria spp.* | **Vitamin E** | 0.28±0.04 | mg/100 g (dw) | Natural sample (fresh), Atlantic coast, Galicia (Spain) | N | Ground, homogenized | Ferraces-Casais et al. (2002) |
| *Porphyra umbilicalis* |  | 0.34±0.11 | mg/100 g (dw) | Natural sample (fresh), Atlantic coast, Galicia (Spain) | N | Ground, homogenized | Ferraces-Casais et al. (2002) |
| *Himanthalia elongata* |  | 2.24±0.05 | mg/100 g (dw) | Natural sample (fresh), Atlantic coast, Galicia (Spain) | N | Ground, homogenized | Ferraces-Casais et al. (2002) |
| *Palmaria palmata* |  | 0.17±0.06 | mg/100 g (dw) | Natural sample (fresh), Atlantic coast, Galicia (Spain) | N | Ground, homogenized | Ferraces-Casais et al. (2002) |
| *Eisenia arborea J.E. Areschoug* |  | 7.58* | mg/100 g (dw) | Natural sample (fresh), Baja California Peninsula, Mexico | N | Sun-dried, ground | Hernández-Carmona et al. (2009) |
| *Tetraselmis suecica* |  | 42.18 | mg/100 g (dw) | Laboratory culture | Unknown | Oven-dried | Fabregas and Herrero (1990) |
| *Isochrysis galbana* |  | 5.82 | mg/100 g (dw) | Laboratory culture | Unknown | Oven-dried | Fabregas and Herrero (1990) |
| *Dunaliella teriolecta* |  | 11.63 | mg/100 g (dw) | Laboratory culture | Unknown | Oven-dried | Fabregas and Herrero (1990) |
| *Chlorella stigmatophora* |  | 66.9 | mg/100 g (dw) | Laboratory culture | Unknown | Oven-dried | Fabregas and Herrero (1990) |
| *Macrocystis pyrifera* | **Vitamin E (α-tocopherol)** | 1327.7± 4.4 | µg/g lipid | Commercial product. Marine Cultivation Company Caldera (Chile) | N | Dried and ground (flour) | Ortiz et al. (2009) |
| *Gracilaria chilensis* |  | 86.5± 2.7 | µg/g lipid | Natural sample (fresh), Natural sample (fresh) Coast, Northern Chile | N | Fresh | Ortiz et al. (2009) |
| *Codium fragile* |  | 453.5 ± 3.2 | µg/g lipid | Natural sample (fresh), Coast, Northern Chile | N | Fresh | Ortiz et al. (2009) |
| *Gracilaria chilensis* | **β-carotene** | 11.37± 0.13 | mg/100 g (dw) | Natural sample (fresh), Coast, Northern Chile | N | Fresh | Ortiz et al. (2009) |
| *Codium fragile* |  | 19.79 ±0.18 | mg/100 g (dw) | Natural sample (fresh), Coast, Northern Chile | N | Fresh | Ortiz et al. (2009) |
| *Tetraselmis suecica* |  | 29.6 | mg/100g (dw) | Laboratory culture | Unknown | Oven-dried | Fabregas and Herrero (1990) |
| *Isochrysis galbana* |  | 7.65 | mg/100g (dw) | Laboratory culture | Unknown | Oven-dried | Fabregas and Herrero (1990) |
| *Dunaliella teriolecta* |  | 8.25 | mg/100g (dw) | Laboratory culture | Unknown | Oven-dried | Fabregas and Herrero (1990) |
| *Chlorella stigmatophora* |  | 0.49 | mg/100g (dw) | Laboratory culture | Unknown | Oven-dried | Fabregas and Herrero (1990) |
| *Caulerpa llentillifera* |  | 9.6 | mg/100 g (dw) | Natural sample (fresh). Hilton Waikaloa, Hawai’i | N | Washed and dried | McDermid and Stuercke (2003) |
| *Codium reediae* |  | 1.62 | mg/100 g (dw) | Natural sample (fresh). RHSF, Hawai’i | N | Washed and dried | McDermid and Stuercke (2003) |
| *Enteromorpha flexuosa* |  | 3.24 | mg/100 g (dw) | Natural sample (fresh). North Shore, O’ahu | N | Washed and dried | McDermid and Stuercke (2003) |
| *Monostroma oxyspermum* |  | 4.2 | mg/100 g (dw) | Natural sample (fresh). Leleiwi, Hawai’i | N | Washed and dried | McDermid and Stuercke (2003) |
| *Ulva fasciata* |  | 10.8 | mg/100 g (dw) | Natural sample (fresh). North Shore, O’ahu | N | Washed and dried | McDermid and Stuercke (2003) |
| *Ulva fasciata* |  | 4.2 | mg/100 g (dw) | Natural sample (fresh), Ma’alaea Bay, Maui | N | Washed and dried | McDermid and Stuercke (2003) |
| *Sargassum echinocarpum* |  | 5.82 | mg/100 g (dw) | Natural sample (fresh), Onekahakaha, Hawai’i | N | Washed and dried | McDermid and Stuercke (2003) |
| *Sargassum obtusifolium* |  | 3.6 | mg/100 g (dw) | Natural sample (fresh), Onekahakaha, Hawai’i | N | Washed and dried | McDermid and Stuercke (2003) |
| *Ahnfeltiopsis concinna* |  | 0.96 | mg/100 g (dw) | Natural sample (fresh), Onekahakaha, Hawai’i | N | Washed and dried | McDermid and Stuercke (2003) |
| *Ahnfeltiopsis concinna* |  | 0.84 | mg/100 g (dw) | Natural sample (fresh), Kona, Hawai’i | N | Washed and dried | McDermid and Stuercke (2003) |
| *Chondrus ocellatus* |  | 1.8 | mg/100 g (dw) | Natural sample (fresh), Leleiwi, Hawai’i | N | Washed and dried | McDermid and Stuercke (2003) |
| *Eucheuma denticulatum* |  | 1.68 | mg/100 g (dw) | Natural sample (fresh), K¯ane’ohe Bay, O’ahu | N | Washed and dried | McDermid and Stuercke (2003) |
| *Gracilaria coropifolia* |  | 0.9 | mg/100 g (dw) | Natural sample (fresh), RHSF, Hawai’i | N | Washed and dried | McDermid and Stuercke (2003) |
| *Gracilaria parvispora* |  | - | mg/100 g (dw) | Natural sample (fresh), KKHH, Moloka‘i | N | Washed and dried | McDermid and Stuercke (2003) |
| *Porphyra vietnamensis* |  | 25.8 | mg/100 g (dw) | Natural sample (fresh), Onekahakaha, Hawai’i | N | Washed and dried | McDermid and Stuercke (2003) |
| *Gracilaria changgi* |  | 5.2±0.4 | mg/100 g (dw) | Natural sample (fresh), Culture Ponds, Fishery Research Institute, Kedah, (Malaysia) | N | Freeze-dried, ground | Norziah and Ching (2000) |
| *Laminaria spp.* |  | 2.20±0.12 | mg/100 g (dw) | Natural sample (fresh), Atlantic coast, Galicia (Spain) | N | Ground, homogenized | Ferraces-Casais et al. (2002) |
| *Porphyra umbilicalis* |  | 3.87±0.50 | mg/100 g (dw) | Natural sample (fresh), Atlantic coast, Galicia (Spain) | N | Ground, homogenized | Ferraces-Casais et al. (2002) |
| *Himanthalia elongata* |  | 4.29±0.11 | mg/100 g (dw) | Natural sample (fresh), Atlantic coast, Galicia (Spain) | N | Ground, homogenized | Ferraces-Casais et al. (2002) |
| *Palmaria palmata* |  | 1.95±0.25 | mg/100 g (dw) | Natural sample (fresh), Atlantic coast, Galicia (Spain) | N | Ground, homogenized | Ferraces-Casais et al. (2002) |
| *Macrocystis pyrifera* |  | 1.74±0.1 | mg/100 g (dw) | Commercial product, Marine Cultivation Company Caldera (Chile) | N | Dried and ground (flour) | Ortiz et al. (2009) |
